# Chronic HDM exposure shows time-of-day and sex-based differences in inflammatory response associated with lung circadian clock disruption

**DOI:** 10.1016/j.isci.2023.107580

**Published:** 2023-08-09

**Authors:** Ashokkumar Srinivasan, Allan Giri, Santhosh Kumar Duraisamy, Alexander Alsup, Mario Castro, Isaac Kirubakaran Sundar

**Affiliations:** 1 Division of Pulmonary, Critical Care and Sleep Medicine, Department of Internal Medicine, University of Kansas Medical Center, Kansas City, KS, USA

**Keywords:** Biological sciences, Immune response, Immunology

## Abstract

Circadian rhythms and sex differences are involved in the pathophysiology of asthma. Yet, there are no reports that simultaneously address the role of the circadian clock and sex-based differences in chronic house dust mite (HDM)-induced asthma. Here, we sought to determine if chronic HDM exposure during the resting phase (zeitgeber time: ZT0/6:00 a.m.) versus the active phase (ZT12/6:00 p.m.) differentially affects the circadian clock and alters asthma pathobiology in female and male mice. HDM exposure at ZT12 exaggerated infiltration of eosinophil subtypes and associated chemokines in females compared to males. Furthermore, HDM exposure augmented eosinophil chemokines, Th2 gene expression and cytokine release, and humoral immune response in females compared to males at ZT12. Concurrently, histopathological evaluation confirmed increased airway inflammation at ZT12 in both females and males. Overall, we showed a time-of-day response and sex-based differences in HDM-induced exaggerated asthmatic phenotypes (inflammation/remodeling) and circadian clock disruption in females compared to males.

## Highlights

• Asthma is a chronic, inflammatory lung disease that shows a time-of-day response
• Chronic HDM exposure during the active phase exhibits sex-based differences
• Th2 cytokines and humoral immune response shows a time-of-day response to allergen
• HDM-induced airway inflammation is associated with lung circadian clock disruption

## Graphical abstract

**Figure:**
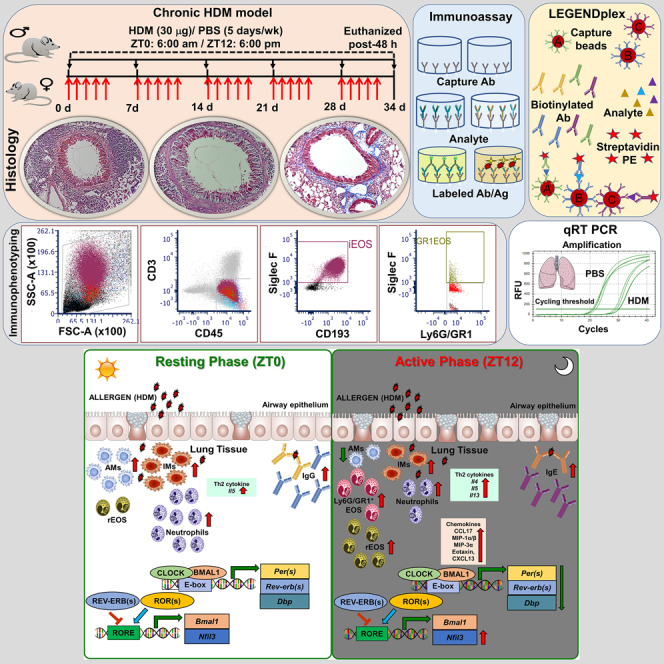

## Introduction

Allergic asthma is a heterogeneous, chronic inflammatory lung disease characterized by the obstruction of airflow and excessive mucus hypersecretion in the airways. House dust mites (HDM) are among the most common household allergen. Intranasal delivery of HDM in mice causes an inflammatory response and remodeling in the lungs. HDM-induced allergic airway inflammation predominantly comprises eosinophils and lymphocytes that mediate the T helper 2 (Th2) immune response. The majority of asthmatics (80% in children and 60% in adults) exhibit a Th2-induced airway inflammation associated with increased blood eosinophilia and IgE-mediated airway hyperresponsiveness (AHR).^1^ Clinically asthma can demonstrate diurnal worsening in the early morning (4:00 a.m.) with symptoms of wheezing, shortness of breath, and chest tightness associated with a decline in forced expiratory volume in 1 s (FEV_1_) and peak expiratory flow rates (PEFR).^2^^,^^3^

Circadian rhythms are intrinsic biological oscillations with a periodicity of nearly 24 h that are maintained in every cell of the body entrained by the circadian clock. The circadian clock is regulated by the CLOCK:BMAL1 complex and clock-related output genes including *Per(s)(Periods 1–3), Cry(s)(Cryptochromes 1–2), Rev-erbα/β (Nr1d1/2), Rorα* and *Nfil3.*^4^^,^^5^ The transcription of these clock genes is tightly regulated by a complex transcriptional-translational feedback loop system. In general, the CLOCK:BMAL1 heterodimer activates the transcription of *Per(s)* and *Cry(s)* during the day. Later, as PER and CRY reach a critical level in the cytosol, they form a heterodimeric protein complex and translocate back to the nucleus to inhibit their transcription by suppressing the CLOCK:BMAL1 complex binding to the E-box promoter. Meanwhile, in the cytosol, PER and CRY are also targeted for ubiquitination and degradation which ensures their gradual decline. This cycle resets approximately every 24 h and is responsible for the observed daily rhythms in physiology and behavior. Another important secondary feedback loop controls *Bmal1* expression through REV-ERB (repressor) and ROR (activator) competing for binding to the retinoic acid orphan receptor element (RORE) within the *Bmal1* promoter and thus fine-tunes the circadian cycles.^4^^,^^5^ A third loop also operates in conjunction with the core and secondary loop involving mainly the D-box binding protein (DBP), which is a repressor of a core circadian clock protein known as the E4 promoter-binding protein 4 (NFIL3). A more detailed description of the lung circadian clock machinery is beyond the scope of this paper but can be found in our recent review.^6^

The circadian clock regulates many physiological processes in the body including the functions of innate and adaptive immunity in response to the changing environment.^7^^,^^8^ In peripheral tissues including the lung, the circadian clock can be altered in response to environmental insults (e.g., allergens, pollens, particulate matter, cigarette smoke [CS], infection, etc.), stressors including odd timings of food intake, and exercise.^9^^,^^10^^,^^11^^,^^12^^,^^13^^,^^14^ The circadian clock regulatory mechanism manifests a time-of-day response following exposure to allergens, CS exposure, and infection.^9^^,^^10^^,^^11^^,^^14^ and therefore pharmacological targeting of the circadian clock may provide better insights into the treatment and management of allergic diseases.^13^^,^^15^^,^^16^^,^^17^

Sex-based dimorphism in asthma prevalence and severity exists. Predominantly, the incidence of asthma is more common and severe in boys than in girls (during early childhood < 5 years), but this trend shifts to a higher prevalence in adolescent women before menopause.^18^^,^^19^^,^^20^^,^^21^ Hence, changes in sex hormones may play a contributing role in the observed sex differences in the pathobiology of asthma. A prior study showed that age and sex-based differences exist in allergen-mediated Th2 response (increased IL-5 and IL-13), total IgE levels and peripheral eosinophil counts in boys at 3 years of age compared to girls.^22^ It is evident that sex differences in immune responses occur throughout life and are influenced by both the age and reproductive status of an individual.^23^ Sex hormone receptors of estrogen and androgen vary throughout the circadian network between males and females from the suprachiasmatic nucleus (SCN) to peripheral organs. This might be due to the sexual dimorphic rhythms of circulating sex hormones including estrogen and testosterone affecting the SCN that manifest through higher amplitude rhythms in female activity patterns compared to males at the behavioral level.^24^^,^^25^ To date, there are no studies that address the complex relationship and difference between time-of-day response and sex-based difference in allergen-induced lung inflammation. In this study, we utilized a chronic HDM-induced asthma model to demonstrate time-of-day response and sex-based differences in the severity of airway inflammation, mucus production, and remodeling associated with circadian clock disruption in the lungs.

## Results

### Chronic HDM exposure shows time-of-day response and sex-based differences in the severity of lung inflammation

C57BL/6 Ncr (WT) mice were exposed to PBS or HDM intranasally for 5 days/week for 5 weeks at a specific zeitgeber time 0 (ZT0/dawn) 6:00 a.m. (resting phase) or ZT12/dusk (6:00 p.m.; active phase) to induce allergic airway inflammation in the lungs. We first analyzed females and males together following chronic HDM exposure at ZT0 and ZT12.

#### HDM-specific changes (combined analysis)

WT mice when analyzed together showed a significant increase in resident eosinophil (**rEOS**: CD11b^+^ SiglecF^+^, and CCR3^-^), inflammatory eosinophil (**iEOS**: CD11b^+^ SiglecF^+^, and CCR3^+^), Ly6G/GR1^+^ Eosinophil (**GR1**^**+**^**EOS**: CD11b^+^ Ly6G/GR1^+^, and SiglecF^+^), interstitial macrophages (IMs), neutrophils, and dendritic cells (DCs) at ZT12 HDM vs. ZT12 PBS. Only for IMs, there was also a significant increase at ZT0 HDM vs. ZT0 PBS (Figure S3).

#### Time-of-day specific changes (combined analysis)

Time-of-day specific increase in rEOS and iEOS were observed at ZT12 HDM vs. ZT0 HDM when males and females were analyzed together. In contrast, DCs were significantly reduced at ZT12 HDM vs. ZT0 HDM when analyzed together (Figure S3). Overall, combined immunophenotyping data in lung tissue suggests that chronic HDM exposure causes an exaggerated immune-inflammatory response (rEOS, iEOS, and DCs) influenced by time-of-day allergen challenge during the active phase (ZT12) vs. the resting phase (ZT0). Next, we separately analyzed females and males following chronic HDM exposure at ZT0 and ZT12.

#### HDM-specific changes (female and male mice)

**Figure 1:**
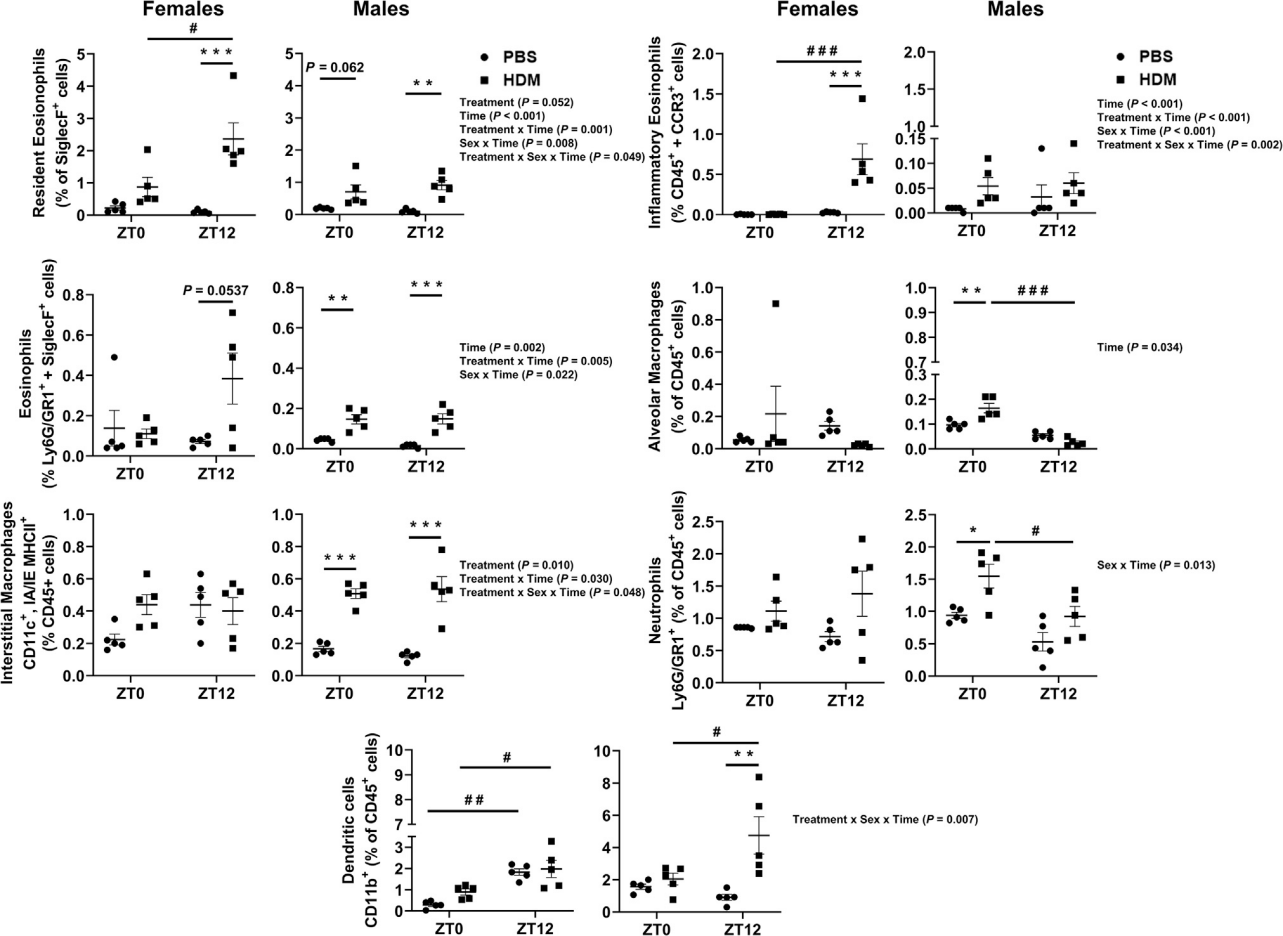
Myeloid cell infiltration shows a time-of-day response and sex-based differences to chronic HDM exposure Myeloid cell types: rEOS, iEos, Gr1^+^Eos, AMs, IMs, neutrophils, and DCs from lung tissues of chronic PBS- and HDM-exposed females and males at ZT0 and ZT12 were analyzed by flow cytometry. Data are shown as mean ± SEM, Two-way ANOVA followed by Tukey’s multiple comparison test (n = 5/group [females and males]). ∗∗p < 0.01, ∗∗∗p < 0.001, compared to respective control (PBS) at ZT0 or ZT12; ^#^p < 0.05, ^# #^p < 0.01, compared to HDM at ZT0 vs. ZT12. Summary statistics for interaction between Treatment x Sex x Time were analyzed using generalized linear modeling using R (see Table S2).

HDM exposure in females significantly increased eosinophil infiltration including all the subtypes (rEOS, iEOS, and Ly6G/GR1^+^EOS) at ZT12 but not at ZT0 when compared to their respective PBS groups (Figure 1). However, in males rEOS and Ly6G/GR1^+^EOS showed a significant increase at both ZT0 and ZT12 when compared to their respective PBS groups with no such difference observed in iEOS (Figure 1). Females showed no difference in alveolar macrophages (AMs), IMs, neutrophils, or DCs at both ZT0 HDM and ZT12 HDM when compared to their respective PBS groups (Figure 1). In contrast, males showed a significant increase in AMs, IMs, and neutrophils at ZT0 HDM vs. ZT0 PBS, and a significant increase in IMs and DCs at ZT12 HDM vs. ZT12 PBS (Figure 1).

#### Time-of-day specific changes (female and male mice)

Time-of-day response in rEOS and iEOS were observed in females with a significant increase at ZT12 HDM vs. ZT0 HDM. However, no time-of-day response was observed for rEOS, iEOS, and Ly6G/GR1^+^EOS in males. Additional interaction analysis showed statistical significance among the three eosinophil subsets (Treatment; Time; Treatment x Time; Sex x Time; Treatment x Sex x Time) (Figure 1; Table S2). Females did not show any time-of-day response to AMs or neutrophils at ZT0 and ZT12 HDM, but males showed a significant reduction in AMs and neutrophils at ZT12 HDM vs. ZT0 HDM. Interaction analysis in AMs showed only statistical significance with Time. IMs did not show time-of-day response in females and males at ZT0 HDM vs. ZT12 HDM. However, interaction analysis in IMs revealed significant changes to Treatment, Treatment x Time, and Treatment x Sex x Time. Interaction analysis in neutrophils showed significant changes with Sex x Time. (Figure 1; Table S2). In both males and females, DCs showed a time-of-day response with a significant increase at ZT12 HDM vs. ZT0 HDM. Interaction analysis in DCs showed statistical significance in Treatment x Sex x Time (Figure 1; Table S2). Overall, females showed a time-of-day response in rEOS, iEOS, and DCs at ZT12 HDM vs. ZT0 HDM. However, males showed a differential time-of-day response in AMs, neutrophils, and DCs in chronic HDM-exposed lungs.

### Chronic HDM exposure shows time-of-day and sex-based differences in chemokine and cytokine gating response in the lung

To determine the time-of-day gating response to chronic HDM-induced chemokines and T helper cytokines in the lung, a flow cytometry-based cytometric bead array was utilized. We first analyzed chemokines in females and males together following chronic HDM exposure at ZT0 and ZT12.

#### HDM-specific changes (combined)

Chemokines including CCL17, MIP-1α, MIP-1β, Eotaxin, and CXCL13 were significantly increased at ZT12 HDM vs. ZT12 PBS, but these chemokines were not affected at ZT0 HDM vs. ZT0 PBS (Figure S4). MIP-3α, KC, and CXCL9 were significantly increased in both ZT0 HDM and ZT12 HDM compared to respective PBS groups (Figure S4). All the remaining chemokines including RANTES (CCL5), MCP-1, IP10, CXCL5, and CCL22 were not affected in the lungs of chronic HDM vs. PBS exposed mice at ZT0 and ZT12 (Figure S5).

#### Time-of-day specific changes (combined)

MIP-1α, Eotaxin, and CXCL13 showed time-of-day gating response with a significant increase at ZT12 HDM vs. ZT0 HDM (Figure S4). T helper cytokine analysis together in females and males revealed significantly increased IL-17A in ZT12 HDM vs. ZT12 PBS with additional Th2 cytokines (IL-4, IL-5, and IL-13) showing trends but not increased significantly at ZT0 or ZT12 HDM compared to respective PBS groups (Table S4). Overall, our combined data analysis (females and males) demonstrates time-of-day chemokine-gating but not specific T helper cytokine-gating response to chronic HDM. Next, we analyzed sex-based differences in chronic HDM-induced lung chemokine gating responses in female and male mice separately.

#### HDM-specific changes (female and male mice)

**Figure 2:**
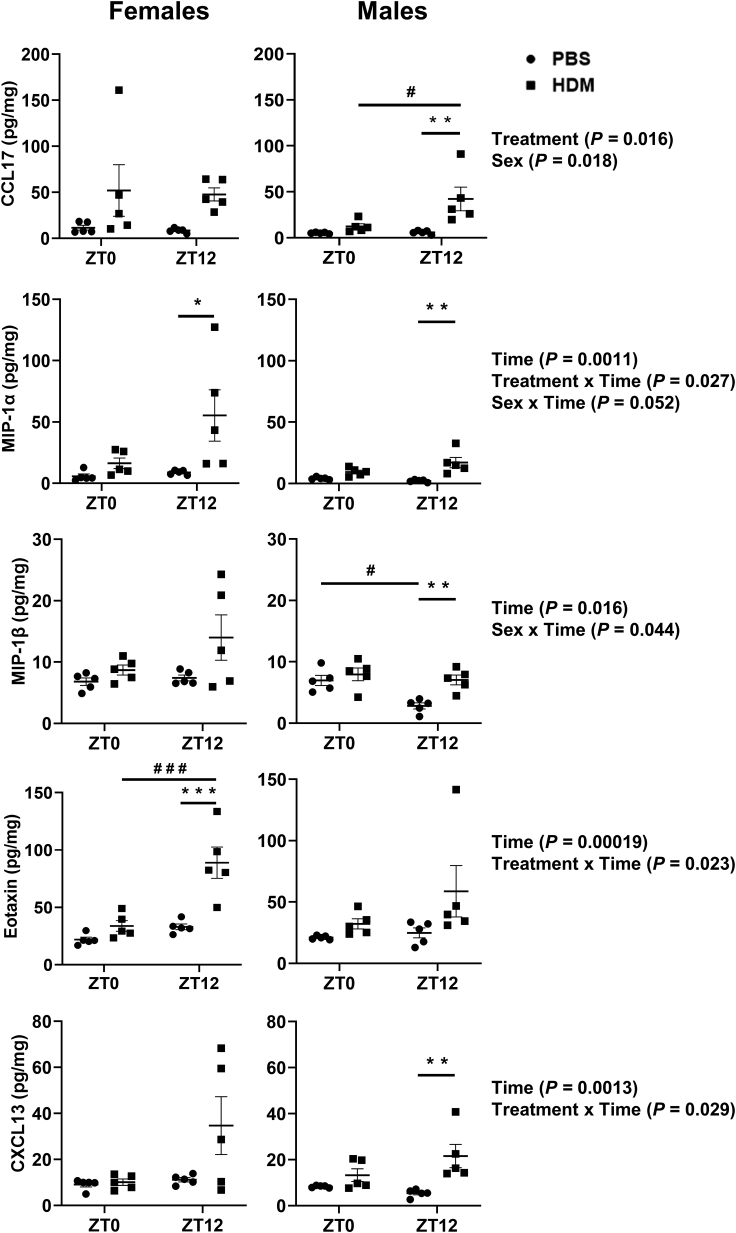
Lung chemokines show a time-of-day response and sex-based differences to chronic HDM exposure Chemokines in lung homogenates of chronic PBS- and HDM-exposed females and males at ZT0 and ZT12 were analyzed using LEGENDplex Mouse Proinflammatory Chemokine Panel (13-plex), based on cytometric bead array method. Two-way ANOVA followed by Tukey’s multiple comparison test (n = 5/group [females and males]). ∗p < 0.05, ∗∗p < 0.01, ∗∗∗p < 0.001, compared to respective control (PBS) at ZT0 or ZT12; ^#^p < 0.05, ^# # #^p < 0.001, compared to PBS or HDM at ZT0 vs. ZT12. Summary statistics for interaction between Treatment x Sex x Time were analyzed using generalized linear modeling using R (see Table S2).

We found that females showed a significant increase in MIP-1α and Eotaxin at ZT12 HDM vs. ZT12 PBS (Figure 2A). CCL17, MIP-1α, MIP-1β, and CXCL13 were significantly increased at ZT12 HDM vs. ZT12 PBS in males (Figure 2B). Chemokine RANTES in females significantly decreased at ZT12 HDM vs. ZT12 PBS but not in males (Figure S6). HDM-exposed females and males showed a significant increase in MIP-3α, and CXCL9 at ZT0 HDM and ZT12 HDM compared to respective PBS groups. Furthermore, females and males showed a significant increase in KC at ZT0 HDM vs. ZT0 PBS, with an increased trend but not a significant change at ZT12 HDM vs. ZT12 PBS (Figure S6). Additionally, females showed a significant increase in CCL22 at ZT12 HDM vs. ZT12 PBS, with a slight increasing trend at ZT0 HDM vs. ZT0 PBS but males remain unaffected at both ZT0 and ZT12 HDM vs. PBS groups (Figure S6). MCP-1, IP-10, and CXCL5 were not significantly affected in females and males at ZT0 and ZT12 HDM vs. PBS (Figure S7).

#### Time-of-day specific changes (female and male mice)

Eotaxin showed a time-of-day gating response at ZT12 HDM vs. ZT0 HDM only in females. CCL17 showed a time-of-day gating response at ZT12 HDM vs. ZT0 HDM only in males (Figure 2). Interaction analysis in lung chemokines revealed statistically significant differences for CCL17 (Treatment; Sex), MIP-1α (Time; Treatment x Time; Sex x Time), MIP-1β (Time; Sex x Time), Eotaxin and CXCL13 (Time; Treatment x Time) (Figure 2; Table S2). Additionally, RANTES showed significant interaction for Treatment x Time, Sex x Time, and Treatment x Sex x Time; but MIP-3α, and CXCL9 showed significant interaction for only Treatment. However, KC showed significant interaction for Treatment x Time. (Figure S6; Table S3). Finally, interaction analysis confirmed statistical significance for MCP-1 (Sex; Sex x Time) and CXCL5 (Sex; Time) even though they were not significant among ZT0 and ZT12 HDM vs. PBS groups (Figure S7; Table S3). We observed trends in increased IL-5, TNFα, IL-4, and IL-17A in females at ZT12 HDM vs. ZT12 PBS that were not statistically significant. However, similar trends were observed only for TNFα and IL-17A in males at ZT0 and ZT12 HDM vs. respective PBS groups. Most of the T helper cytokines analyzed in lung homogenates were not statistically significant in females and males exposed to chronic HDM (Table S4). Overall, our findings support that HDM-exposed females and males show differential time-of-day gating responses in lung chemokines and Th2 cytokines associated with exaggerated airway inflammation.

### Chronic HDM exposure shows time-of-day and sex-based differences in Th2 cytokine gene expression in the lungs

To determine the role of time-of-day response in the gene expression of Th2 cytokines (*il4*, *il5*, and *il13)* that drive chronic HDM-induced allergic airway inflammation qRT-PCR analysis was performed. We analyzed gene expression of Th2 cytokines in females and and males together following chronic HDM exposure at ZT0 and ZT12.

#### HDM-specific changes and Time-of-day specific changes (combined)

Time-of-day response was observed in *il5* and *il13* expression at ZT12 HDM vs. ZT0 HDM (Figure S8). These findings support that both *il5* and *il13* may be the key Th2 cytokines driving chronic HDM-induced recruitment and activation of eosinophil subsets differentially at ZT12 vs. ZT0 in the lungs. Next, we analyzed the gene expression of Th2 cytokines in females and males exposed to chronic HDM separately.

#### HDM-specific changes (female and male mice)

**Figure 3:**
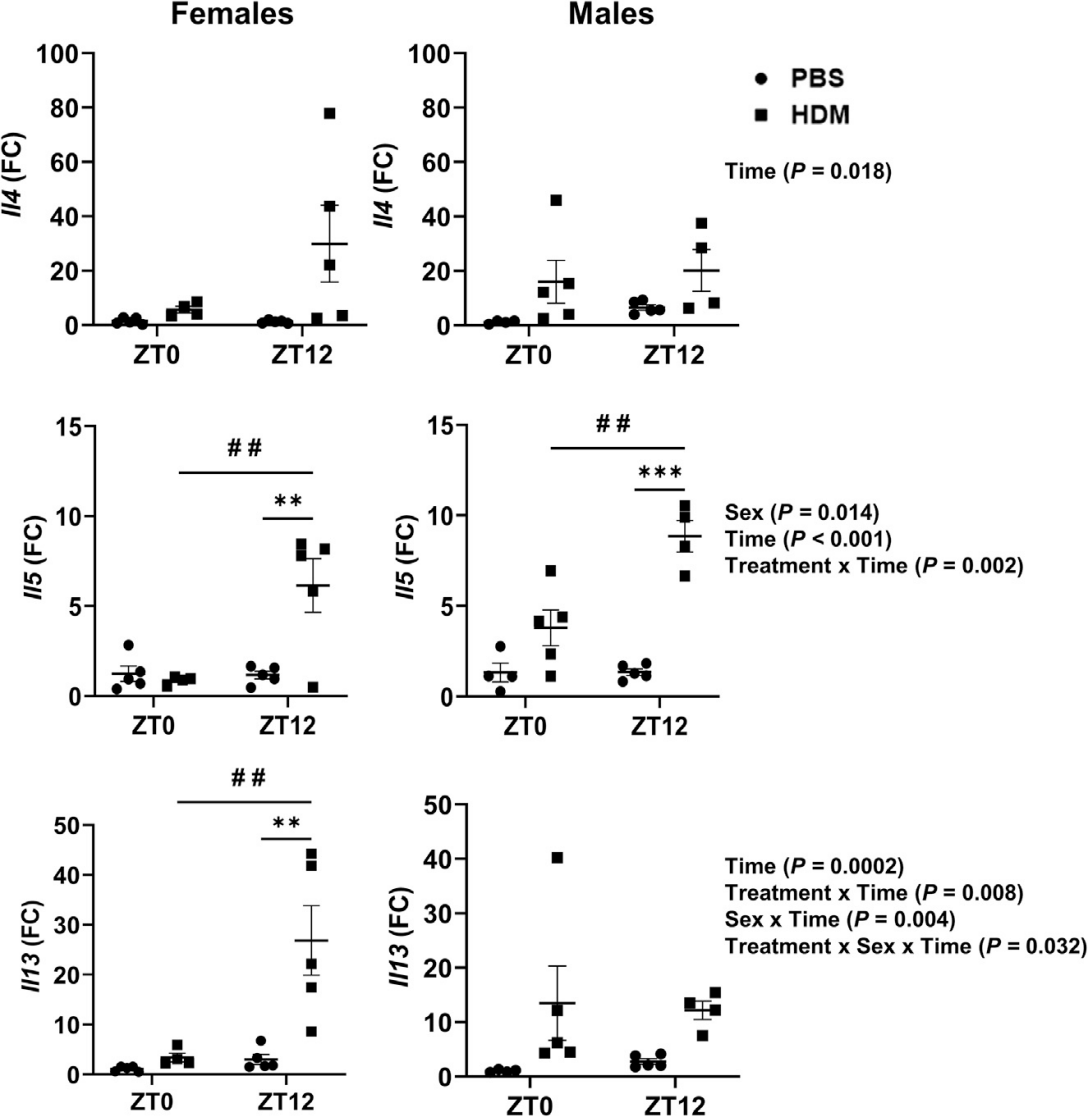
Gene expression of Th2 cytokines shows a time-of-day response and sex-based differences to chronic HDM exposure Total RNA was isolated from the lungs of chronic PBS- and HDM-exposed females and males at ZT0 and ZT12. Gene expression of Th2 chemokines (*il4*, *il5*, and *il13*) were determined by qRT-PCR analysis relative to 18S rRNA (*Rn18S*) as housekeeping control. Relative expression (fold change) was determined by the 2^−ΔΔCt^ method. Two-way ANOVA followed by Tukey’s multiple comparison test (n = 5/group [females and males]). ∗∗p < 0.01, ∗∗∗p < 0.001, compared to respective control (PBS) at ZT0 or ZT12; ^#^^#^p < 0.01, compared to HDM at ZT0 vs. ZT12. Summary statistics for interaction between Treatment x Sex x Time were analyzed using generalized linear modeling using R (see Table S2).

In females, a significant increase in the expression of *il5* and *il13* was observed at ZT12 HDM vs. ZT12 PBS, but not at ZT0 HDM vs. ZT0 PBS (Figure 3) There was a trend in increased expression of *il4*, *il5*, and *il13* observed in males at ZT0 and ZT12 HDM vs. PBS, but significant for *il5* at ZT12 HDM vs. ZT12 PBS (Figure 3)

#### Time-of-day specific changes (female and male mice)

Females showed time-of-day response with a significant increase in *il5* and *il13* at ZT12 HDM vs. ZT0 HDM. However, the transcript levels for all three Th2 cytokines were not affected at ZT0 HDM vs. ZT0 PBS in females (Figure 3). Similarly, Th2 cytokine genes in males showed a time-of-day gating response for only *il5* at ZT12 HDM vs. ZT0 HDM (Figure 3). Interaction analysis revealed statistically significant differences for *il4* (Time), *il5* (Sex; Time; Treatment x Time), and *il13* (Time; Treatment x Time; Sex x Time; Treatment x Sex x Time) gene expression (Figure 3; Table S2). Our data support that the time-of-day response and sex-based difference in the severity of lung inflammation observed may be due to altered expression of *il5* and *il13* in the lung.

### Chronic HDM exposure shows time-of-day and sex-based differences in total and HDM-specific immunoglobulins

We determined the time-of-day response in serum total (IgE and IgG) and HDM-specific immunoglobulins (IgE, IgG, IgG1, IgG2b, IgM, and IgA) utilizing ELISA.

#### HDM-specific changes (combined)

When males and females were analyzed together total IgE and IgG were significantly elevated at ZT0 and ZT12 HDM vs. PBS groups (Figure S9). HDM-specific immunoglobulin levels including IgG, IgG1, IgG2b, IgM, and IgA were significantly increased at ZT0 and ZT12 HDM compared to respective PBS groups. However, HDM-specific IgE was increased at ZT12 HDM vs. ZT12 PBS but not at ZT0 HDM vs. ZT0 PBS (Figure S10).

#### Time-of-day specific changes (combined)

When females and males were analyzed together total IgE levels were significantly greater at ZT12 HDM vs. ZT0 HDM (Figure S9). HDM-specific IgG response correlated inversely with the HDM-specific IgE response (significantly higher at ZT0 HDM vs. ZT12 HDM) (Figure S10). Overall, we found that chronic HDM exposure differentially affects both total and HDM-specific immunoglobulins at ZT12 vs. ZT0. Next, we analyzed the time-of-day response and sex-based difference in serum total and HDM-specific immunoglobulins in chronic HDM-exposed male and female mice separately.

#### HDM-specific changes (female and male mice)

**Figure 4:**
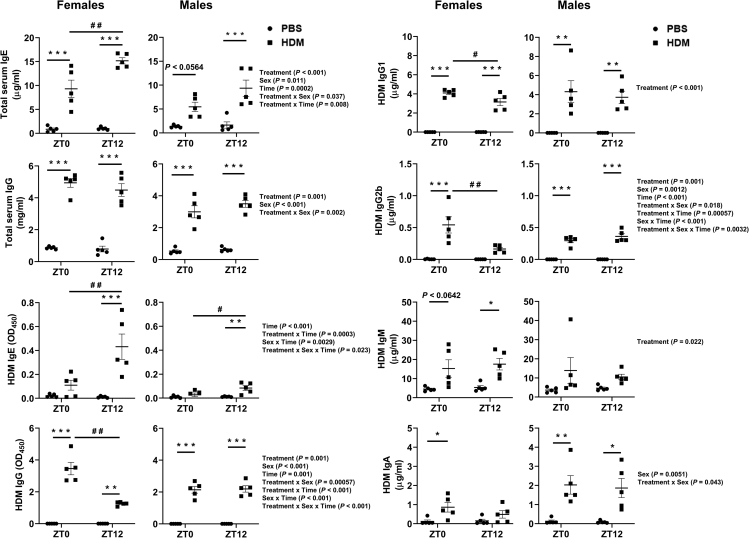
Serum levels of total and HDM-specific immunoglobulins show a time-of-day response and sex-based differences to chronic HDM exposure Total IgE, Total IgG, HDM-specific IgE, HDM-specific IgG, HDM-specific IgG1, HDM-specific IgG2b, HDM-specific IgM, and HDM-specific IgA in the serum of chronic PBS- and HDM-exposed females and males at ZT0 and ZT12 were determined by ELISA. Data were expressed as ng/ml and mg/ml for total IgE and total IgG, respectively. HDM-specific immunoglobulins (IgE, IgG, IgG1, IgG2b, IgM, and IgA) in the serum were determined by commercially available ELISA kits (Chondrex, Inc.). Unable to detect HDM-specific IgG2a and IgG3 levels in PBS- and HDM-exposed mice. Data for the HDM-specific immunoglobulins were expressed as absorbance at 450 nm or μg/ml. Data are shown as mean ± SEM, Two-way ANOVA followed by Tukey’s multiple comparison test (n = 5/group). ∗p < 0.05, ∗∗p < 0.01, ∗∗∗p < 0.001, compared to respective control (PBS) at ZT0 or ZT12; ^#^p < 0.05, compared to HDM at ZT0 vs. ZT12; ^# #^p < 0.01, compared to HDM at ZT0 vs. ZT12. Summary statistics for interaction between Treatment x Sex x Time were analyzed using generalized linear modeling using R (see Table S2).

Females and males showed a significant increase in both total serum IgE and IgG at ZT0 and ZT12 HDM compared to their respective PBS groups (Figure 4). Interestingly, HDM-specific IgE in females increased only at ZT12 HDM but not at ZT0 HDM compared to their respective PBS groups. Other HDM-specific immunoglobulins including IgG, IgG1, and IgM in females increased at both ZT0 and ZT12 HDM vs. PBS groups. However, only HDM-specific IgA and IgG2b showed no changes in the immunoglobulin level at ZT12 HDM vs. ZT12 PBS (Figure 4). A similar trend was also observed in males where HDM-specific IgE increased only at ZT12 HDM but not at ZT0 HDM compared to their respective PBS groups (Figure 4). Other HDM-specific immunoglobulins in males including IgG, IgG1, IgG2b, and IgA all increased significantly at both ZT12 and ZT0 HDM when compared to their respective PBS groups. Only HDM-specific IgM showed no significant increase at ZT0 and ZT12 HDM compared to their respective PBS groups (Figure 4).

#### Time-of-day specific changes (female and male mice)

Females showed a significant time-of-day response in total IgE at ZT12 HDM vs. ZT0 HDM but not for total IgG. Interaction analysis showed significant changes for total IgE (Treatment; Sex; Time; Treatment x Sex; Treatment x Time) and total IgG (Treatment; Sex; Treatment x Sex) (Figure 4; Table S2). Sex-based comparison of HDM-specific immunoglobulins in females revealed a time-of-day response to IgE, IgG, IgG1, and IgG2b antibodies in ZT12 HDM vs. ZT0 HDM. Interestingly, HDM-specific IgE was significantly increased at ZT12 HDM in females and males compared to ZT0 HDM, and this effect was more prominent in females. Interaction analysis showed significant differences among Time, Treatment x Time, Sex x Time, and Treatment x Sex x Time for HDM-specific IgE (Figure 4; Table S2). Additionally, HDM-specific IgG subtypes (IgG, IgG1, and IgG2b) were significantly increased in females at ZT0 HDM vs. ZT12 HDM, but not in males. Interaction analysis revealed statistical significance for HDM-specific IgG and IgG2b (Treatment; Sex; Time; Treatment x Sex; Treatment x Time, Sex x Time; Treatment x Sex x Time). Similarly, interaction analysis showed only Treatment response for HDM-specific IgG1 and IgM. Finally, interaction analysis for HDM-specific IgA showed a significant difference in Sex and Treatment x Sex (Figure 4; Table S2). Overall, we noted a significant time-of-day and sex-based difference in total and HDM-specific immunoglobulin response to chronic HDM exposure.

### Chronic HDM exposure does not affect the stress hormones corticosterone and serotonin

#### HDM-specific changes

We found no significant difference in serum corticosterone levels between PBS and HDM groups at both ZT0 and ZT12 when females and males were analyzed together or separately. Similarly, serotonin levels were neither affected in the combined analysis nor when females and males were analyzed separately at ZT0 and ZT12 HDM vs. PBS (Figures S11 A and B).

#### Time-of-day specific changes

We observed an increase in serum corticosterone levels at ZT12 PBS vs. ZT0 PBS but not with HDM-exposed females and males analyzed together (Figure S11A). However, when females were analyzed separately, corticosterone was significantly increased at both ZT12 PBS and ZT12 HDM compared to ZT0 PBS and ZT0 HDM, respectively. Males did not show time-of-day response to corticosterone levels when analyzed separately (Figure S11B). Interaction analysis revealed significant differences in serum corticosterone levels in Time and Sex x Time. However, no time-of-day response in serotonin levels was observed in both females and males (Figure S11; Table S3). Together, these findings suggest that the stress hormone corticosterone may have partly influenced time-of-day response in females but not in males during chronic HDM exposure.

### Chronic HDM exposure shows time-of-day response and sex-based differences in lung inflammation, mucus production, and collagen deposition

To determine the difference in lung immune-inflammatory response that correlates with histopathological evaluation in chronic HDM-exposed mice, we analyzed different compartments of the lung (airway/peribronchial, perivascular, and alveolar regions) for inflammation, mucus production, and collagen deposition.

#### HDM-specific changes

**Figure 5:**
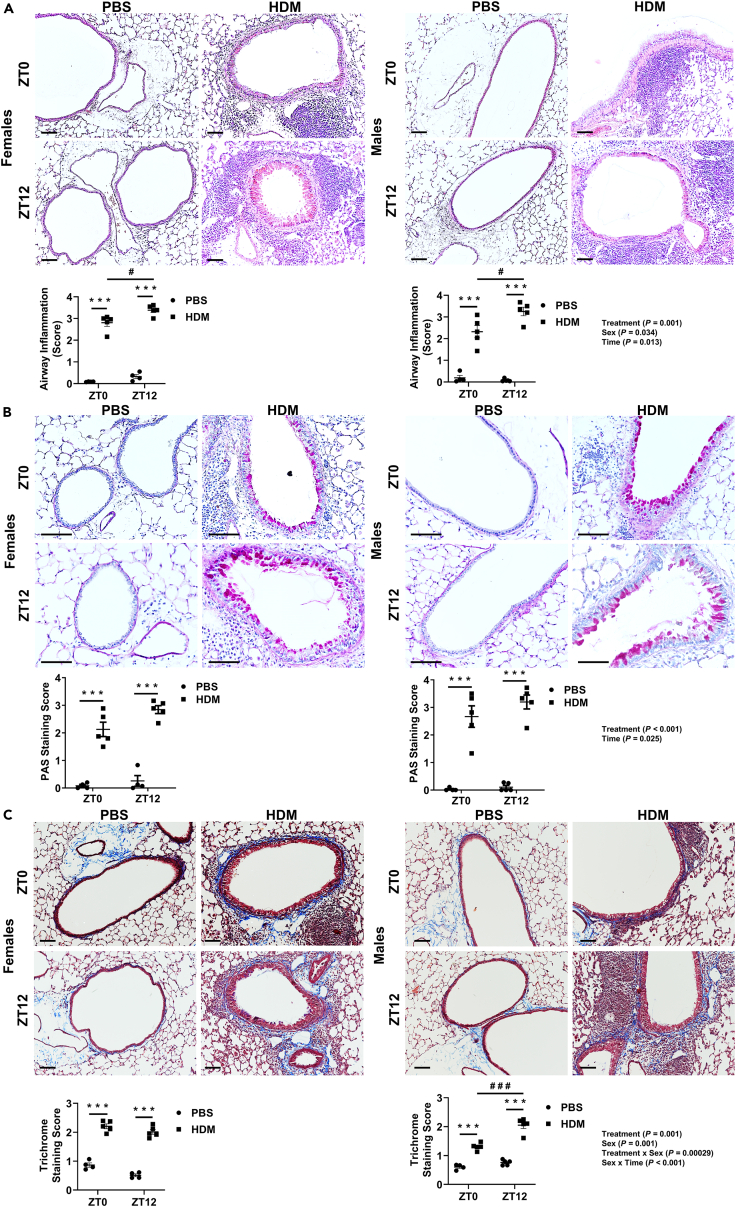
The histological evaluation shows a time-of-day response and sex-based differences in the degree of lung airway inflammation, mucus production, and collagen deposition to chronic HDM exposure (A) Representative Hematoxylin and Eosin (H&E) stained lung tissue sections showing a difference in the degree of lung airway inflammation. (B) Representative Periodic-Acid Schiff’s-stained lung tissue sections showing a difference in mucus production. (C) Representative Trichome stained lung tissue sections showing a difference in extracellular matrix accumulation/collagen deposition from chronic PBS- and HDM-exposed females and males at ZT0 and ZT12. The graph shows the average lung airway inflammation scores from different regions, mucus staining scores, and trichrome staining scores performed using the scoring criteria (see STAR Methods section) in a blinded manner. Scale bar (100 μm). Data are shown as mean ± SEM, two-way ANOVA followed by Tukey’s multiple comparison test (n = 4–5/group [females and males]). ∗∗∗p < 0.001, compared to respective control (PBS) at ZT0 or ZT12; ^#^p < 0.05, compared to HDM at ZT0 vs. ZT12; ^# # #^p < 0.001, compared to HDM at ZT0 vs. ZT12. Summary statistics for interaction between Treatment x Sex x Time were analyzed using generalized linear modeling using R (see Table S2).

Airway/bronchial, perivascular, and alveolar inflammation quantified by H&E staining was significantly increased in both females and males at ZT0 and ZT12 HDM compared to respective PBS groups (Figure 5A; Figures S12; and S13A and B). Periodic-Acid Schiff staining for mucus production and collagen deposition confirmed by Trichome staining showed a significant increase at ZT0 and ZT12 HDM vs. PBS in both females and males (Figures 5B and 5C).

#### Time-of-day specific changes

We found a significant time-of-day response with increased airway and alveolar inflammation, and mucus production, at ZT12 HDM vs. ZT0 HDM when females and males were analyzed together (Figure S12). No time-of-day difference was observed for mucus production in both males and females analyzed separately. Interaction analysis for mucus production revealed a significant difference in Treatment and Time response (Figure 5B; Table S2). H&E staining revealed severe airway/bronchial inflammation showing time-of-day response in females and males at ZT12 HDM vs. ZT0 HDM. Interaction analysis for airway inflammation revealed significant differences among Treatment, Sex, and Time (Figure 5A; Table S2). Males showed a time-of-day response in increased alveolar inflammation at ZT12 HDM vs. ZT0 HDM but not in females (Figure S13B). This was also true for collagen deposition where a time-of-day response was only observed in males showing a significant increase at ZT12 HDM vs. ZT0 HDM. Interaction analysis for collagen deposition data showed Treatment, Sex, Treatment x Sex, and Sex x Time were statistically significant (Figure 5C; Table S2). Interaction analysis of perivascular inflammation data showed Treatment and Time response. Finally, alveolar inflammation data showed significant interaction for Treatment, Sex, Time, Treatment x Sex, and Sex x Time (Figure S13; Table S3). Overall, histopathological evaluation supports that time-of-day and sex-based differences influence the lung immune-inflammatory response following chronic HDM exposure.

### Chronic HDM exposure differentially affects circadian clock gene expression in a sex-dependent manner

We analyzed circadian clock gene expression utilizing qRT-PCR following chronic HDM exposure in mice.

#### HDM-specific changes

**Figure 6:**
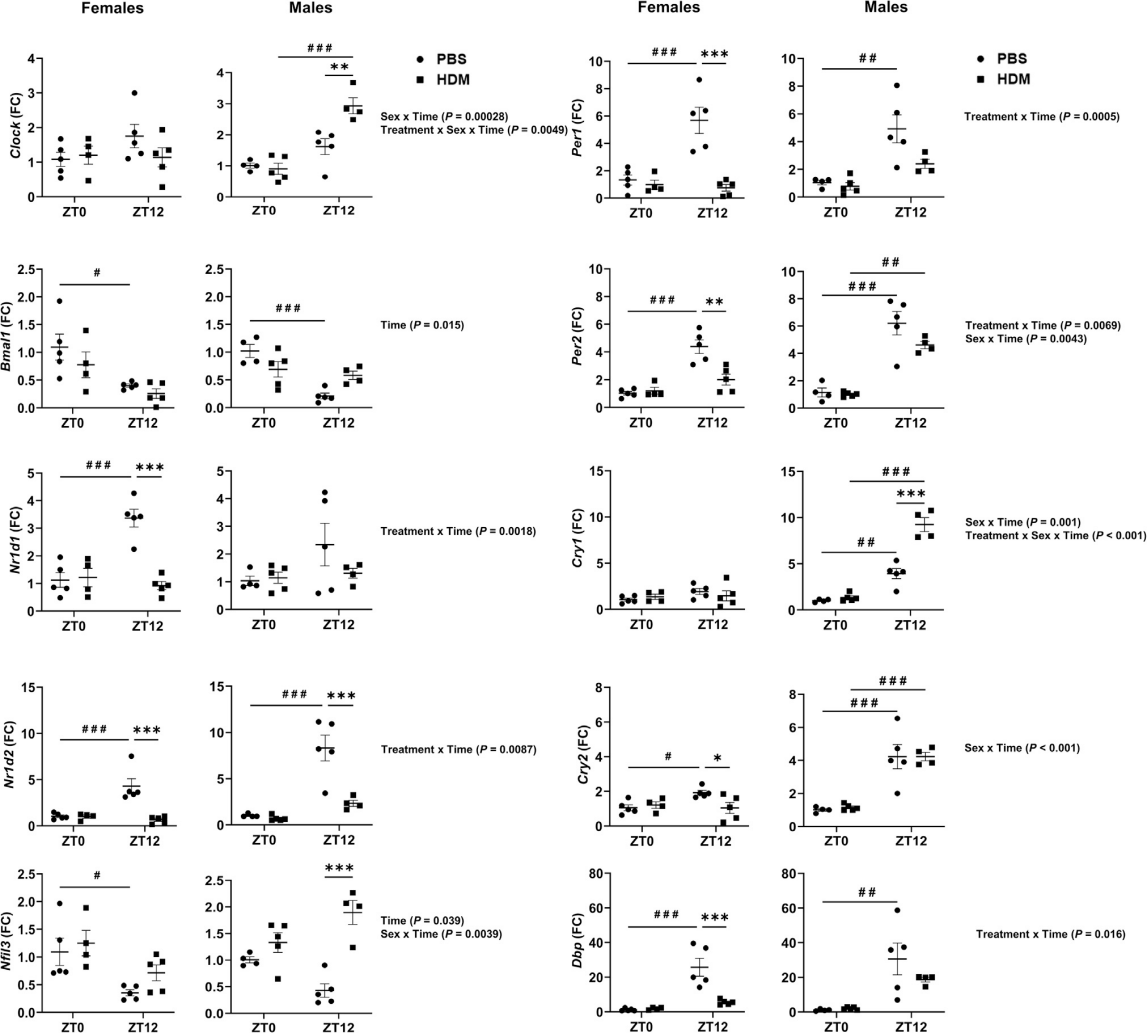
Gene expression of circadian clock genes show a time-of-day response and sex-based differences to chronic HDM exposure Total RNA was isolated from the lungs of chronic (5 days/week for 5 weeks) PBS- and HDM-exposed females and males at ZT0 and ZT12. Gene expression of core clock-controlled genes (*Clock*, *Bmal1*, *Nr1d1*, *Nr1d2*, *Nfil3*, *Per1*, *Per2*, *Cry1*, *Cry2*, and *Dbp*) were determined by qRT-PCR analysis relative to 18S rRNA (*Rn18S*) as housekeeping control. Relative expression (FC: fold-change) was determined by the 2^−ΔΔCt^ method. Data are shown as mean ± SEM, Two-way ANOVA followed by Tukey’s multiple comparison test (n = 4–5/group [females and males]). ∗p < 0.05, ∗∗p < 0.01, ∗∗∗p < 0.001, compared to respective control (PBS) at ZT0 or ZT12; ^#^p < 0.05, ^# #^p < 0.01, ^# # #^p < 0.001, compared to PBS or HDM at ZT0 vs. ZT12.Summary statistics for interaction between Treatment x Sex x Time were analyzed using generalized linear modeling using R (see Table S2).

Interestingly, clock gene expression was not significantly altered at ZT0 HDM vs. ZT0 PBS for any of the core clock genes including *Clock*, *Bmal1*, *Nr1d1*, *Nr1d2*, *Nfil3*, *Per1*, *Per2. Cry1*, *Cry2*, *Dbp*, and *Rora* when females and males were analyzed together (Figures S14 and S15). These results were consistent at ZT0 HDM vs. ZT0 PBS even when females and males were analyzed separately (Figure 6). However, gene expression of a few core clock genes including *Nr1d1*, *Nr1d2*, *Per1*, *Per2*, and *Dbp* was significantly reduced when analyzed together at ZT12 HDM vs. ZT12 PBS (Figure S14). Females showed a significantly reduced expression of *Nr1d1*, *Nr1d2*, *Per1*, *Per2*, *Cry2*, *Dbp*, and *Rora*, while males showed a significant increase of *Clock*, *Nfil3*, *Cry1*, and a significant reduction in *Nr1d2* only at ZT12 HDM vs. ZT12 PBS (Figures 6 and S15)*.*

#### Time-of-day specific changes

When females and males were analyzed together, *Bmal1* was significantly reduced at ZT12 in both PBS and HDM groups compared to ZT0 PBS and HDM, respectively. There was no significant difference between ZT0 HDM and ZT12 HDM compared to respective PBS groups for *Bmal1.* Similarly, as expected, *Nr1d1* and *Nr1d2* were significantly increased at ZT12 PBS compared to ZT0 PBS when analyzed together (Figure S14). However, this difference was abolished in the HDM group where no difference was observed between ZT12 HDM compared to the ZT0 PBS and HDM groups. *Clock* gene remained unaffected at both ZT0 and ZT12 PBS and HDM, and *Nfil3* expression was not affected at ZT0 PBS and HDM but slightly increased at ZT12 HDM vs. PBS when analyzed together (Figure S14). Interestingly, we observed that core clock genes *Per2* and *Cry1* significantly increased at ZT12 HDM vs. ZT0 HDM when analyzed together hinting that the time of allergen exposure differentially affects clock gene expression at the transcriptional level. *Per1* and *Cry2* showed an expected trend at ZT10 vs. ZT12 PBS (increased), but no such difference in gene expression was observed at ZT12 HDM compared to ZT0 PBS (Figure S14).

Sex-based differences revealed an interesting pattern in the expression of core clock genes in females compared to males. In females, gene expression of *Bmal1* and *Nfil3* was decreased and *Nr1d1*, *Nr1d2*, *Per1*, *Per2*, and *Cry2* were increased at ZT12 PBS vs. ZT0 PBS However, this was completely abolished by HDM exposure. No significant change in the expression of core clock genes was observed between ZT0 HDM vs. ZT12 HDM (Figure 6). Males showed augmented gene expression of *Clock*, *Bmal1*, *Per2*, *Cry1*, and *Cry2* at ZT12 HDM vs. ZT0 HDM, although the fold changes were quite different. Thus, the results from our study highlight that females may be more susceptible to circadian clock disruption in the lungs following HDM exposure. Gene expression of *Clock* and *Cry1* remained unaffected in the females at both ZT0 and ZT12 PBS and HDM (Figure 6). Interaction analysis revealed differential responses that were statistically significant for *Clock* and *Cry1* (Sex x Time; Treatment x Sex x Time), *Bmal1* (Time), *Nr1d1*, *Nr1d2*, *Per1*, and *Dbp* (Treatment x Time), *Nfil3* (Time; Sex x Time), *Per2* (Treatment x Time; Sex x Time). Finally, *Cry2* and *Rora* showed significant interaction for Sex x Time response (Figures 6 and S15; Tables S2 and S3). Overall, these findings support the altered expression of core clock genes in the lungs of chronic HDM-exposed female and male mice.

## Discussion

The focus of our current study was to determine if chronic HDM-induced airway inflammation, mucus production, and extracellular matrix remodeling in the lung were influenced by the time of HDM exposure and whether there are sex-based differences in mice. Interestingly, we found that the mice challenged with HDM at ZT12 (active phase/dark cycle) has significantly more eosinophilic infiltrates in their lungs compared to those challenged at ZT0 (resting phase/light cycle). Previous studies have reported that dendritic cells promote Th2-mediated eosinophilic inflammation in response to inhaled allergen.^26^ Our findings corroborate that dendritic cells drive eosinophilic airway inflammation at ZT12 HDM in the combined (females and males) analysis.^26^^,^^27^ Surprisingly, even though males had higher DCs in the lungs, the proportion of eosinophils was relatively lesser compared to females. Meanwhile, females displayed relatively lower DCs in the lung, but higher eosinophils compared to males. The important interleukins (IL-4 and IL-13) that induce the expression of eosinophilic adhesion molecules such as VCAM-1 either displayed a higher trend (for IL-4) or was significantly higher (IL-13) in females but not in males.^28^ Thus, we showed that DCs may not be the sole contributing factor driving eosinophilic inflammation.

Eosinophil recruitment is tightly regulated by specific chemokines and Th2 cytokines in the lungs during chronic HDM-induced allergic asthma. Among the spectrum of chemokines studied, Eotaxin-1 and RANTES are potent chemoattractants for eosinophils from the blood to the site of inflammation. Eotaxin is more selective toward eosinophils when compared to RANTES, even though they both share the same receptor (CCR3).^29^^,^^30^ In this study, we noticed Eotaxin levels were increased in females at ZT12 HDM associated with an exaggerated infiltration of eosinophils. This strongly suggests that eosinophilic infiltration in the lung at ZT12 is a chemoattractant-driven response and may not be due to a simply increased population of eosinophils in the bone marrow and blood alone following HDM exposure. In contrast, the chemokine CCL17 was significantly increased in males at ZT12 HDM, which is primarily released by DCs to attract monocytes and Th2 lymphocytes.^31^^,^^32^ We believe that this increase in DCs observed in males at ZT12 HDM may be responsible for the heightened CCL17 levels in the lungs. MIP-1α, MIP-1β, and CXCL13 are the other chemokines elevated in males at ZT12 HDM. Chemokines MIP-1α and MIP-1β exhibit similar functions, as they both attract monocytes, DCs, and neutrophils by binding to CCR1 and CCR5 receptors, respectively.^33^ These findings are consistent with lung immunophenotyping analysis of HDM-exposed male mice showing increased IMs, rEOS, Ly6G/GR1^+^ EOS, and DCs. In contrast, eosinophil subsets were increased in female mice at ZT12 HDM, indicating that specific chemokines and Th2 cytokines drive response to allergic airway inflammation in a sex-dependent manner. The observed differential time-of-day response to lung chemokines following chronic HDM exposure may be due to altered circadian clock gene expression in the lungs. Additionally, sex-based differences that influence the recruitment of myeloid immune cell types disproportionately but would require circadian gene knockout (KO) mice studies to further confirm this response.

Previous studies have shown that chronic HDM exposure via the intranasal route in mice augments Th2-mediated airway inflammation and remodeling.^34^^,^^35^ Particularly, IL-5 produced by Th2 has been long associated with several allergic diseases including rhinitis and asthma. At the mRNA level, *il5* expression was significantly increased in both females and males at ZT12 HDM vs. ZT12 PBS, but not at ZT0. The increased expression of *il5* in the lung at ZT12 supports our observation of higher eosinophils as it is known to prolong survival and enhance effector function.^28^ On the other hand, an increased mRNA expression of *il13* at ZT12 HDM was only found in female mice. IL-13 is well-known for its role in mediating AHR and pathophysiological changes during chronic asthma. This includes goblet cell hyperplasia (mucus hypersecretion), airway smooth muscle proliferation, and subepithelial fibrosis. In addition, IL-13 also promotes the B cell antibody class switch to IgE.^36^ The increased mRNA expression of *il13* in ZT12 HDM-exposed females was associated with significantly higher total IgE levels and exaggerated airway inflammation, and remodeling compared to males at ZT12.

Total and HDM-specific IgE levels in response to chronic HDM confirm our previous observation that female mice show a greater degree of lung inflammation compared to males due to an overall increase in the expression of Th2 cytokines. Unlike total IgE, the total serum level of IgG did not show any time-of-day response or sex-based difference. Interestingly, HDM-specific serum IgG subtypes (IgG, IgG1, and IgG2b) showed an inverse phenotype of what was observed in serum IgE levels (increased at ZT0 HDM vs. ZT12 HDM in females). HDM-specific IgG antibodies have been reported to decrease allergic airway inflammation, therefore higher HDM-specific IgG levels at ZT0 HDM in females partly explain why they show comparatively less airway inflammation. Other serum immunoglobulins IgM and IgA did not show any time-of-day response in HDM-exposed females but were slightly higher in ZT0 and ZT12 in response to HDM. We found male mice showed greater HDM-specific IgA levels compare to HDM-exposed female mice. IgA is a mucosal antibody that has been reported to reduce secondary allergen sensitization/exposure.^37^ This may be one of the possible reasons for male mice showing relatively lesser overall airway inflammation and associated lung phenotypes compared to HDM-exposed female mice.

*Nr1d1/2* circadian clock gene expression was significantly reduced at ZT12 HDM vs. ZT12 PBS in our study. NR1D1 holds significant importance in regulating innate immune response and immune cell trafficking during inflammation.^38^^,^^39^ A prior report showed that HDM exposure in WT mice at the active phase (Dark cycle/Dusk) significantly increased the magnitude of AHR compared to HDM-exposed mice during the resting phase (Light cycle/Dawn). However, *Rev-erbα* KO mice exposed to HDM either during the active phase or resting phase abolished the time-of-day response supporting the potential role of circadian clock *Rev-erbα* in HDM-induced AHR.^10^ *Nr1d1* also plays a crucial role in reducing Th2 differentiation as well as the formation of Th2 cells by binding to the GATA binding protein 3 (GATA3) gene promoter. GATA3 is very essential for Th2 differentiation and transcription of Th2 cytokines in Th2 cells. Therefore, NR1D1 binding to GATA3 results in decreased expression of Th2 cytokines. *Rev-erbα* KO shows elevated Th2 cells and related cytokines indicating the protective role of NR1D1 in allergic asthma.^40^ Results from our study support the possible role of *Nr1d1/2* (reduced mRNA expression) in chronic HDM-induced allergic asthma with heightened *il4*, *il5*, and *il13* expression at ZT12. However, we believe that altered clock genes in the lungs during asthma are a result of complex molecular interaction between transcriptional-translational regulation of circadian clock molecules (at the mRNA and protein level) and other key transcription factors which needs further investigation.

The functional role of PERs in immune regulation remains unclear and nothing is known in terms of asthma pathobiology. A study showed that intense light therapy improved the alveolar barrier function and dampened lung inflammation caused by *Pseudomonas aeruginosa*-induced acute lung injury and alveolar type 2 (AT-2) cell-specific *Per2* deletion abolished the protective response in mice.^41^ Furthermore, *Per1* KO mice displayed altered rhythms of interferon-γ cytokine, and cytolytic factors (perforins and granzyme B) at the gene and protein level in splenic natural killer (NK) cells indicating *Per1* gene regulation through NK cell clock to modulate immune function in mice.^42^ Accumulating evidence suggests that Th1 cytokine such as interferon-γ reduces airway response to allergen challenge by counteracting the Th2 response during allergic asthma.^43^ In the present study, HDM-induced immune-inflammatory response reduced *Per1/2* expression in the lung. However, how the reductions in the PER proteins contribute to a Th2 dominant response in asthma remains inconclusive from our study. Future studies are needed to delineate how disruption of core clock proteins such as PER1/2/3 can cause an imbalance in the type of T cell response (Th1 vs. Th2).

Chronic HDM exposure have a major impact on the expression of *Cry1/2*. Notably, *Cry2* expression was significantly reduced in females at ZT12 HDM, and *Cry1/2* expression was increased in males at ZT12 HDM demonstrating sex-based differences. Even though cryptochrome genes are not directly involved in Th2-mediated inflammation, *Cry1/2* double KO cells show activation of NF-κB and protein kinase signaling pathways thereby enhancing the susceptibility to chronic inflammatory diseases.^44^ Another report showed adenovirus-mediated overexpression of CRY1 in mice blocked inflammatory cytokines (IL-6, TNFα, and IL-1β) and adhesion molecule (VCAM-1, ICAM-1, and E-selectin) expression including NF-κB activation (Phospho-p65).^45^ These findings corroborate well with our results where relatively lower *Cry1* and significantly reduced *Cry2* expression at ZT12 HDM in females is associated with increased airway inflammation. On the other hand, increased *Cry1* expression in males may help explain the relatively milder inflammatory phenotypes.

DBP (transcriptional activator) and NFIL3 (repressor) are antagonistic in their function, where they competitively bind to the D-box promoter region to regulate *Per(s)* and *Ror(s)* genes. DBP role in asthma remains unexplored. However, NFIL3 on the other hand is known for its anti-inflammatory role in asthma. It is expressed at low levels in natural killer cells, B cells, T cells, DCs, and macrophages and activated by specific cytokines IL-3, IL-4, IL-10, and hormones including glucocorticoids (GCs).^46^ NFIL3 suppresses IL-5 and IL-13 expression by directly binding to the promoter of respective genes. NFIL3 in B cells promotes IgE class switching when stimulated with IL-4.^47^^,^^48^ An earlier report showed the mechanism of GC-induced apoptosis resistance in activated eosinophils was mediated by Pim-1-induced NFIL3 that was enhanced by glucocorticoid receptor (GR) transactivation (TA). Additionally, blocking the Pim-1/NFIL3 axis or selective elimination of GR-mediated TA restores apoptosis in IL-5-activated eosinophils suggesting NFIL3’s role in treating eosinophilic disorders associated with steroid resistance.^49^ In this study, we found that *Nfil3* expression was not significantly affected in HDM-exposed groups at ZT0 and ZT12 (combined analysis). However, *Nfil3* transcript levels were increased in males at ZT12 HDM which correlates with lower Th2 cytokines and reduced IgE levels relative to females at ZT12 HDM. Small molecules that target NFIL3 can be of potential benefit to attenuate HDM-induced allergic airway inflammation in females with and without steroid-resistant asthma phenotype. In contrast to *Nfil3* expression, transcript levels of *Dbp* were significantly reduced in females compared to males at ZT12 HDM. Based on the close functional relationship between NFIL3 and DBP, it is safe to assume they may be significant candidate genes to devise options for treating patients with severe asthma.

Recent discoveries have highlighted the role of type-2 innate lymphoid cells (ILC2s) in asthma pathophysiology. ILC2s produce many Th-cell-associated cytokines including IL-5 and IL-13. While IL-5 induces eosinophil infiltration, IL-13 stimulates mucus hyperproduction resulting in exaggerated type 2 inflammation in the airway. Furthermore, some studies have reported female mice having a significantly higher number of ILC2s than males due to the inhibitory effect of androgen signaling on ILC2s development. However, this difference in number has not always been the case.^50^ In our study, we did not quantify ILC2 which may have contributed significantly to the observed differences noted in this study. There are both environmental as well as intrinsic factors that influence the ILC2 function in the lung. This includes circadian rhythm, sex hormones, age, and neurotransmitters.^51^ Future studies should attempt to investigate the role of ILC2 in the pathophysiology of asthma and determine if there is a time-of-day difference in the lung ILC2, which can further validate the time-dependent effects we observed in our current study.

Eosinophils develop in bone marrow from hematopoietic stem cells, which give rise to the eosinophil progenitors during hematopoiesis. Previous studies have already confirmed that eosinophils increase in number in bone marrow, blood, and BAL of mice during allergic diseases.^52^ However, we did not quantify the eosinophil number in bone marrow or blood. It might be interesting to observe how eosinophil number might vary in the blood following chronic HDM exposure at ZT12 vs. ZT0. This might help us to further confirm if the exaggerated eosinophil infiltration we observed at ZT12 was a chemoattractant-driven response or a time-dependent increase in eosinophil number in the blood.

The genetic backgrounds of mice are important determinants of allergic airway inflammation. T cells from C57BL/6 mice preferentially produce Th1 cytokine with high interferon-γ (IFNγ) and low IL-4, whereas those from BALB/c mice favor Th2 cytokine production (IL-4, IL-5) with low IFNγ and high IL-4. Thus, C57BL/6 and BALB/c mice are, respectively, regarded as Th1- and Th2-dominant mouse strains widely used as preclinical models of allergic asthma.^53^^,^^54^ We only utilized C57BL/6 WT mice in this study. Prior studies using BALB/c mice showed robust eosinophil and neutrophil infiltration in response to HDM.^55^ Another report is conflicting that showed no such differences in airway inflammation between BALB/c and C57BL/6 mice.^56^ Herein, we observed an exaggerated eosinophilic response using the C57BL/6 strain, despite being a Th1-biased mouse strain. It is well-known in the literature that Th1 cells antagonize Th2 functioning which plays a central role in sustaining asthmatic response in the lung. Since Th2 cells mediate the activation of the humoral immune response against allergens, we speculate that using BALB/c strain with a Th2 bias would likely augment eosinophilic infiltration in HDM-exposed female mice.

AHR is a characteristic feature of asthma that shows a distinct diurnal variation.^10^ AHR is an exaggerated obstructive response to a variety of inhaled stimuli such as methacholine or allergen and is often used clinically for the diagnosis of asthma. In our study, we observed time-of-day differences in several parameters though we did not measure AHR. It would have been interesting to observe how time-of-day affects AHR in males and females separately, and how this contributes to the exaggerated eosinophil infiltration observed in our study.

Given the sex-based differences in HDM exposure in exaggerated infiltration of eosinophils, higher eosinophil chemokines, and Th2 cytokine as well as gene expression in females compared to males, it is important to consider if sex hormones are among the key driving factor for the observed differences. Previous studies have shown that ovariectomy inhibits lung eosinophilia and IL-5 levels in the lung when exposed to ovalbumin (OVA). Eosinophilic responses to OVA were partly restored when estradiol was reintroduced in those ovariectomized mice.^57^ The phases of the estrous cycle combined with the timing of the HDM exposure (ZT0 vs. ZT12) may affect the outcomes and should be an important parameter to consider when conducting future studies using female mice. Future studies may attempt to investigate how conducting the same study as ours but using ovariectomized mice may change the outcomes.

In summary, we show time-of-day and sex-based differences in the immune-inflammatory, humoral, chemokine/cytokine-gating response to chronic HDM-induced asthma. HDM exposure during the dark/active phase (ZT12) in mice showed exaggerated asthmatic phenotypes in females compared to males. The time-dependent increase in eosinophilic inflammation, chemokine/cytokine release, along with total and HDM-specific IgE antibody response was associated with repressed expression of core clock genes and pathological outcomes such as airway inflammation, mucus production, and collagen deposition/remodeling in the lungs. Overall, the results from our study highlight the need to conduct circadian clock gene deletion (global) and lung cell type-specific KO studies that may provide a better understanding of the observed time-of-day response on phenotypes in allergic asthma models. Future studies should aim to address how specific clock genes (e.g., *Nr1d1* and *Nfil3*) play an essential role in asthma pathophysiology using chronotherapeutic approaches to delineate observed sex-based differences in severe asthma.

### Limitations of the study

In our study, we utilized lung tissues instead of BAL fluid for immunophenotyping analysis from female and male mice exposed to either PBS or HDM at ZT0 and ZT12. The observed increase in eosinophils following chronic HDM exposure may be a result of increased eosinophil production in the bone marrow or simply a chemoattractant-driven response, investigating which was beyond the scope of the current study. We focused entirely on WT mice. However, simultaneously comparing WT with certain circadian gene knockout models such as *Rev-erbα* KO mice may help better understand the causal effect relationship of time-of-day response as well as the sex-based difference in immune-inflammatory response, humoral response, and circadian clock disruption in HDM-induced allergic asthma. Furthermore, our study did not include sex-based difference at the lung transcriptome (RNA-seq) level that affects inflammation and associated canonical pathways. Transcriptomic changes in specific clock genes may help determine if the observed results from our study truly correlate with the protein abundance in the lungs and their localization using target-specific antibodies/probes. We have discussed other intriguing aspects such as the role of ILC2, hematopoietic stem cells, and mouse genetic background including other aspects of the sex-based difference in asthma pathobiology. Future studies should aim to delineate some of the key molecular mechanisms driving lung circadian clock disruption (global vs. cell type-specific deletion of circadian genes) resulting in pathophysiological outcomes following chronic HDM-induced allergic asthma.

## STAR★Methods

### Key resources table

**Table:** 

REAGENT or RESOURCE	SOURCE	IDENTIFIER
**Antibodies**		

Purified anti-mouse CD16/32 antibody (clone 93)	BioLegend	Cat# 101302; RRID:AB_312801
Ghost Dye Violet 510	TonboBio	Cat# 13-0870-TT100
Brilliant Violet 605 anti-mouse CD45 antibody (clone 30-F11)	BioLegend	Cat# 103155; RRID:AB_2650656
PR PE/Cyanine7 anti-mouse CD3ε antibody (clone 145-2C11)	BioLegend	Cat# 100320; RRID:AB_312685
APC/Cyanine7 anti-mouse/human CD11b antibody (clone M1/70)	BioLegend	Cat# 101226; RRID:AB_830642
Brilliant Violet 650 anti-mouse I-A/I-E antibody (clone M5/114.15.2)	BioLegend	Cat# 107641; RRID:AB_2565975
Brilliant Violet 785 anti-mouse CD11c antibody (clone N418)	BioLegend	Cat# 117336; RRID:AB_2565268
Alexa Fluor 488 anti-mouse CD24 antibody (clone M1/69)	BioLegend	Cat# 101816; RRID:AB_493482
PE anti-mouse CD193 (CCR3) antibody (clone J073E5)	BioLegend	Cat# 144506; RRID:AB_2561534
Alexa Fluor 700 anti-mouse Ly-6G antibody (clone 1A8)	BD Biosciences	Cat# 561236; RRID:AB_10611860
APC anti-mouse CD64 (FcγRI) antibody (clone X54-5/7.1)	BioLegend	Cat# 139306; RRID:AB_11219391
PE-CF594 rat anti-mouse Siglec-F (clone E50-2440)	BD Biosciences	Cat# 562757; RRID:AB_2687994

**Chemicals, peptides, and recombinant proteins**		

House Dust Mite Extract 25 ml VI B82 (Lot # 371587)	Greer Laboratories, Inc.	Cat# XPB82D3A25ML
Liberase TM Research Grade	Sigma-Aldrich	Cat# 5401119001
TRIzol Reagent	Invitrogen	Cat# 15596026
DNase I recombinant, RNase-free	Sigma Aldrich	Cat# 4716728001
Stain Buffer (FBS)	BD Biosciences	Cat# 554656; RRID:AB_2869006

**Critical commercial assays**		

LEGENDplex Mouse Proinflammatory chemokine panel (13 plex)	BioLegend	Cat# 740007
LEGENDplex Mouse T Helper Cytokine panel (12 plex)	BioLegend	Cat# 741044
Mouse IgG ELISA kit	Bethyl Laboratories	Cat# E99-131; RRID:AB_2892028
Mouse IgE ELISA kit	Bethyl Laboratories	Cat# E99-115; RRID:AB_2892026
Mouse Anti-House Dust Mite (HDM) IgA Antibody Assay Kit	Chondrex, Inc.	Cat# 3046
Mouse Anti-House Dust Mite (HDM) IgE Antibody Assay Kit	Chondrex, Inc.	Cat# 3047
Mouse Anti-House Dust Mite (HDM) IgG Antibody Assay Kit	Chondrex, Inc.	Cat# 3030
Mouse Anti-House Dust Mite (HDM) IgG1 Antibody Assay Kit	Chondrex, Inc.	Cat# 3034
Mouse Anti-House Dust Mite (HDM) IgG2a Antibody Assay Kit	Chondrex, Inc.	Cat# 3038
Mouse Anti-House Dust Mite (HDM) IgG2b Antibody Assay Kit	Chondrex, Inc.	Cat# 3035
Mouse Anti-House Dust Mite (HDM) IgM Antibody Assay Kit	Chondrex, Inc.	Cat# 3036
Serotonin ELISA kit	Enzo Life Sciences, Inc.	Cat# ADI-900-175
Corticosterone ELISA kit	Enzo Life Sciences, Inc.	Cat# ADI-900-097
Periodic Acid-Schiff (PAS) kit	Sigma-Aldrich	Cat# 395B-1KT
Trichrome Stain (Masson) kit	Sigma-Aldrich	Cat# H15-1KT
RNeasy Mini kit	Qiagen	Cat# 74104
OneComp eBeads Compensation Beads	ThermoFisher Scientific	Cat# 01-1111-42
LS columns	Miltenyi Biotech	Cat# 130-042-401
CD326 (EpCAM) MicroBeads, mouse	Miltenyi Biotech	Cat# 130-105-958; RRID:AB_2936423

**Experimental models: Organisms/strains**		

Mouse: C57BL/6NCr (Wild-type)	NCI (Frederick, MD)	Strain Code: 556

**Oligonucleotides**		

RT-qPCR primers (see Table S1)	This paper	N/A

**Software and algorithms**		

FCS Express 7	*De Novo* Software	https://denovosoftware.com
R statistical computing language (version 4.2.3; R Core Team 2021)	R Foundation for Statistical Computing (Vienna, Austria)	https://www.R-project.org/

### Resource availability

#### Lead contact

Further information and request for resources, data and reagents should be directed to and will be fulfilled by the lead contact, Isaac K. Sundar (isundar@kumc.edu).

#### Materials availability

This study did not generate new unique reagents.

### Experimental model and study participant details

#### Animals

The animal experiments in this study were conducted as per the National Institute of Health, Guide for the care and use of laboratory animals as approved by the Institutional Animal Care and Use Committee at the University of Kansas Medical Center (ACUP No. 2020–2575). Adult C57BL/6NCr (Wild-type [WT], females and males, 8 weeks old, Strain Code: 556) were obtained from National Cancer Institute (Frederick, MD) and housed in a regular 12; 12 light-dark cycle, with *ad libitum* access to food and water at the Research Support Facility (RSF) vivarium in the University of Kansas Medical Center.

#### Chronic house dust mite (HDM) model

Adult C56BL/6NCr female and male mice were sensitized/challenged with sterile phosphate-buffered saline (PBS control) and HDM (30 μg/30 μl; intranasally) (Greer Laboratories, Lenoir, NC; Lot # 371587) under 5% mild isoflurane anesthesia at specific time-of-day [Zeitgeber time ZT0: 6:00 am (dawn/lights on/resting phase) or ZT12: 6:00 pm [dusk/lights off/active phase)] for 5 days/week for a total of 5 weeks (Figure S1). C57BL/6NCr is a substrain of C57BL/6 founder line C57BL/6NCr also known as Ly5.1 strain. This C57BL/6NCr strain is currently available from the National Cancer Institute (NCI, Frederick) through the vendor NCI Charles River ideally used for inflammation and immunological adoptive transfer research.^58^^,^^59^ All the parameters were measured 48 h post-last PBS/HDM challenge in mice at ZT0 or ZT12.

### Method details

#### Lung digestion

The right middle lobe of the lung was minced into small pieces and resuspended in DMEM F12 media (5 ml) containing 2.6 IU Liberase (Sigma-Aldrich) and 30 μg DNase I (Sigma-Aldrich). Tissue digestion was carried out under constant shaking at 37°C for 1 h. After incubation, the single cell suspension was made by pipetting up and down followed by filtering the cells through 70 micron and 40 micron nylon mesh to remove clumped cells and undigested tissues. Depletion of lung epithelial cells in single cells suspension was carried out using CD326 (EpCAM) MicroBeads (Miltenyi Biotec) and magnetically sorted through the LS column (Miltenyi Biotec). The single cell suspension devoid of lung epithelial cells was washed and counted using the Countess 3 Automated cell counter. The dissociated lung cells were resuspended in 100 μl of BD stain buffer (BD Bioscience) containing 1.0 × 10^6^ cells and blocked with anti-CD16/32 antibody before staining to block non-specific binding of staining antibody (BioLegend, San Diego CA).

#### Flow cytometry analysis

Dissociated lung cells were resuspended in 100 μL staining buffer and blocked with anti-CD16/32 antibody (BioLegend) followed by staining using fluorescently-tagged antibodies: anti-CD45 BV605 (BioLegend), anti-CD3 PE-Cy7 (BioLegend), anti-CD11b (BioLegend), anti-IA/IE BV650 (BioLegend), anti-CD11c (BioLegend), anti-CD24 AF488 (BioLegend), anti-CD193 PE (BioLegend), anti-GR1 AF700 (BioLegend), anti-CD64 APC (BioLegend), anti-Siglec-F PE-CF594 (BD Bioscience), along with a viability indicator Ghost Dye Violet 510 (Tonbo Bioscience). The list of antibodies, sources, and identifier (Cat#) information is summarized in the key resources table. The data acquisition was performed in LSRII Fortessa by collecting a total of 100,000 events (∼90% single cell events/samples) and analyzed using FCS Express 7 software. Dead cells were excluded based on Ghost dye^+^ (dead cells) and Ghost dye¯ live cells were included for further gating. Compensation for each flow cytometer experiment was performed with unstained and all single-color controls using OneComp eBeads (Thermo Fisher Scientific). Representative dot plots of a multicolor flow cytometry panel used to identify myeloid cell subsets in lung tissues from mice were provided as described (Figure S2).^60^

#### Chemokine analysis in lung tissues

Lung tissue ∼50 mg was homogenized in PBS (500 μl) containing Halt protease inhibitor (Thermo Scientific) and centrifuged at 2000 rpm for 10 min. Lung homogenates (supernatant) were stored at −80°C until use. Chemokine analysis in lung homogenates was performed using the LEGENDplex mouse proinflammatory chemokine panel (BioLegend) according to the manufacturer’s protocol. Samples were run on Attune NxT flow cytometer and data were analyzed using the LEGENDplex data analysis suite (BioLegend).

#### Total IgE and IgG ELISA

The total IgE and IgG levels in serum samples were measured using ELISA kits (Bethyl Laboratories, Inc. TX, US) according to the manufacturer’s protocol. Serum samples were diluted at 1:20 and 1:100,000 for total IgE and IgG, respectively. In brief, 96 well plates were coated with anti-IgE/IgG capture antibody and blocked for 2 h followed by the addition of diluted serum samples to respective wells as mentioned above. After 1 h incubation, plates were washed thrice with 1X PBS containing Tween 20 (0.05%). Then, a biotin-conjugated detection antibody was added and incubated for 1 h followed by washing (3 times) and the addition of streptavidin-HRP to the plate and incubated for 30 min. After the final wash steps, Tetramethylbenzidine (TMB) substrate was added and incubated for 30 min. The color reaction was stopped using 2N sulfuric acid. Using the endpoint kinetic method, the color intensity was measured at 450 nm which directly correlates with the standard concentration. Values were expressed as ng/ml for total IgE and mg/ml for total IgG.

#### HDM-specific immunoglobulins ELISA

HDM-specific IgE, IgA, IgM, IgG, IgG_1_, and IgG2b antibodies in serum were measured using ELISA kits (Chondrex, Inc.). Sample dilution for all the HDM-specific immunoglobulins are as follows: IgE (1:10), IgA (1:20), 1:10 (IgM), and IgG, IgG1, and IgG2b (1:100). HDM-specific IgE was measured using 96 well plate coated with anti-mouse IgE and HDM-specific IgE were detected using biotinylated HDM and the results were expressed as absorbance at 450 nm. For all the other HDM-specific antibodies in serum, HDM-coated 96 well plates were used using standard sandwich ELISA according to the manufacturer’s instructions.

#### Corticosterone and serotonin ELISA

Corticosterone and serotonin levels in the serum of chronic HDM exposed mice were analyzed using ELISA kits (Enzo Life Sciences, Inc) with 1:40 and 1:32 sample dilutions, respectively. Both corticosterone and serotonin were measured using a competitive immunoassay according to the manufacturer’s protocol. An alkaline phosphatase and para-nitrophenyl phosphate (PNPP) detection system was used, and the reaction was stopped using trisodium phosphate solution. The optical density of samples was measured at 405 nm which inversely correlates with the absorbance of standards.

#### Gene expression profiling using quantitative real-time PCR (qRT-PCR)

Lung tissues collected from mice challenged with HDM and PBS control were snap frozen and stored at −80°C. Fifty milligrams of lung tissue were homogenized in Trizol and total RNA was isolated using the RNeasy kit (Qiagen), according to the manufacturer’s instructions. The extracted total RNA was quantitated using a Nanodrop spectrophotometer (Nanodrop One; Thermo Scientific). cDNA was prepared with 1 μg of total RNA using the First Strand cDNA synthesis kit (Qiagen) and stored at −20°C which was subsequently used for quantitative real-time polymerase chain reaction (qRT-PCR). For normalization 18S ribosomal RNA (*Rn18s*) was used as a housekeeping control.^61^ The CT value for 18S ribosomal RNA was subtracted from the CT value for the gene of interest to obtain a delta CT (ΔCT) value. The relative fold-change for each gene was calculated using the 2^−ΔΔCt^ method as described.^61^^,^^62^ The list of mouse gene-specific qRT-PCR primers used in the study is provided (Table S1).

#### Lung histology

The left larger lobe of the lung was inflated with 1% low melting agarose in 1X PBS. The inflated lung tissues were fixed in 10% neutral buffered formalin for 48 h. Lung tissues were progressively dehydrated using ethanol (30%, 50%, and 70% respectively; for 20 min each). Paraffin-embedded midsagittal lung tissues were sectioned using a microtome (5 μm sections) to reveal the longitudinal view of the main intrapulmonary bronchus of the left lobe. The lung sections were stained with H&E, Periodic Acid-Schiff (PAS), and Masson trichrome staining for histopathological scoring and analysis.

#### Hematoxylin and eosin (H&E), periodic acid-schiff (PAS) and trichome staining

H&E staining was used to determine the compartment-specific inflammation status of lung tissues (peri-bronchial [airways], vascular [perivascular], and alveolar). For all histopathological analyses, a semi-quantitative unbiased scoring approach was used by blinding the experimental groups. In brief, we used a five-point scoring scheme, score 0: indicates normal lung with no infiltration of inflammatory cells; score 1: indicates less inflammation containing a few immune cells around the region of interest; score 2: indicates mild inflammation with a few layers of immune cells around the region of interest; score 3: indicates moderate inflammation with additional 3–5 layers of immune cells around the region of interest; score 4: indicates exaggerated inflammation surrounded by several layers of immune cells around the region of interest. Several sections from each experimental group were analyzed, and the mean inflammation scores were used to determine the statistical significance.

PAS staining was performed using a PAS staining kit (Sigma-Aldrich) according to the manufacturer’s instructions. We used a specific scoring criterion (0–4) to assess mucin production in the airway epithelium: score 0: indicates 0.5% PAS-positive cells (relatively no staining in the airway epithelium); score 1: indicates 25% PAS-positive cells; score 2: indicates 25–50% PAS-positive cells; score 3: indicates 50–75% PAS-positive cells and score 4: indicates greater than 75% PAS-positive cells. Several sections from each experimental group were analyzed, and the means PAS scores were used to determine the statistical significance.

Trichome staining was performed using a Trichome staining kit according to the manufacturer’s instructions. We used defined scoring criteria (0–4) to determine collagen deposition around the peri-bronchial region. Score 0: indicates normal lung, score 1: indicates very less collagen deposition with no thickening around the bronchial vessel, score 2: indicates mild collagen deposition accompanied by slight thickening around the peri-bronchial region, score 3: indicates moderate collagen deposition associated with slightly increased thickening around the per-bronchial region and around the vessels, score 4: indicates high collagen deposition accompanied by thickening of the peri-bronchial region and the vessels. Several sections from each experimental group were analyzed, and the mean Ashcroft’s scores were used to determine the statistical significance.

### Quantification and statistical analysis

All the parameters obtained from different experimental groups (ZT0 PBS, ZT0 HDM, ZT12 PBS, and ZT12 HDM) were analyzed by GraphPad Prism 9 software (La Jolla, CA). Results were analyzed using Two-way ANOVA followed by Tukey’s multiple comparison test. Each p value is adjusted to account for multiple comparisons. Three-way interaction analysis between Treatment (PBS vs. HDM), Sex (Females vs. Males), and Time (ZT0 vs. ZT12) was assessed using the generalized linear modeling through the R statistical computing language. For each outcome measure, a generalized linear model was fit including Two-way and Three-way interactions. Due to the limited sample size of the experiment and in consideration of convergence issues, this analysis is included as supplementary tables and descriptive statistics within each of the revised figures Data were expressed as the mean ± SEM with a p value <0.05 was defined as statistically significant.

## Data Availability

• Data present in this manuscript will be shared by the corresponding author upon request. • This paper does not report original code. Any additional information required to reanalyze the data reported in this paper is available from the lead contact upon request.

## Supplemental information

Document S1. Figures S1–S15 and Tables S1, S4 and S5

Table S2. Two-way and Three-way interaction analysis and P-value for data are presented in the main figures (Main Figures 1–6) of this study

Table S3. Two-way and Three-way interaction analysis and P-value for data are presented in supplementary figures (Figures S6, S7, S11, S13, and S15) of this study
