# Supplementary material for: Chronic HDM exposure shows time-of-day and sex-based differences in inflammatory response associated with lung circadian clock disruption
Source: iScience. 2023 Aug 9;26(9):107580. doi: 10.1016/j.isci.2023.107580 (PMC10470299; doi:10.1016/j.isci.2023.107580)
Supplement: Document S1. Figures S1–S15 and Tables S1, S4 and S5 [file mmc1.pdf]

iScience, Volume *26*

Supplemental information

Chronic HDM exposure shows time-of-day and sex-based differences in inflammatory response associated with lung circadian clock disruption

Ashokkumar Srinivasan, Allan Giri, Santhosh Kumar Duraisamy, Alexander Alsup, Mario Castro, and Isaac Kirubakaran Sundar

Schematic representation of chronic HDM-induced allergic asthma

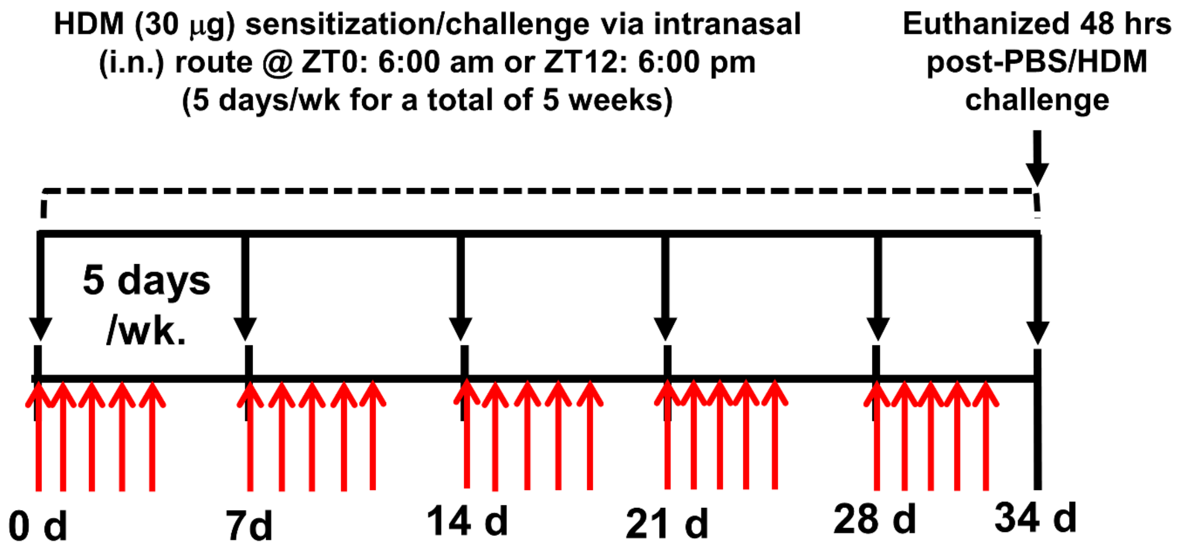

Figure S1. Schematic representation of chronic HDM-induced allergic asthma model used in this study; related to Figures 1-6. C57BL/6J (2-3 months old, female and male) mice were sensitized/challenged via the intranasal (i.n.) route with house dust mite extract (HDM: 30 µg in 30 µL) or sterile 1X PBS control (30 µL) at ZT0/ZT12 (Zeitgiber time 0 or 6:00 am [dawn/resting phase] or Zeitfiber time 12 or 6:00 pm [dusk/active phase] for 5 days/week for a total of 5 weeks under mild anesthesia using 5% isoflurane. Serum, BAL fluid, and lung tissues were collected 48 hours post-last exposure for flow cytometry, histopathology, and biochemical and molecular analysis.

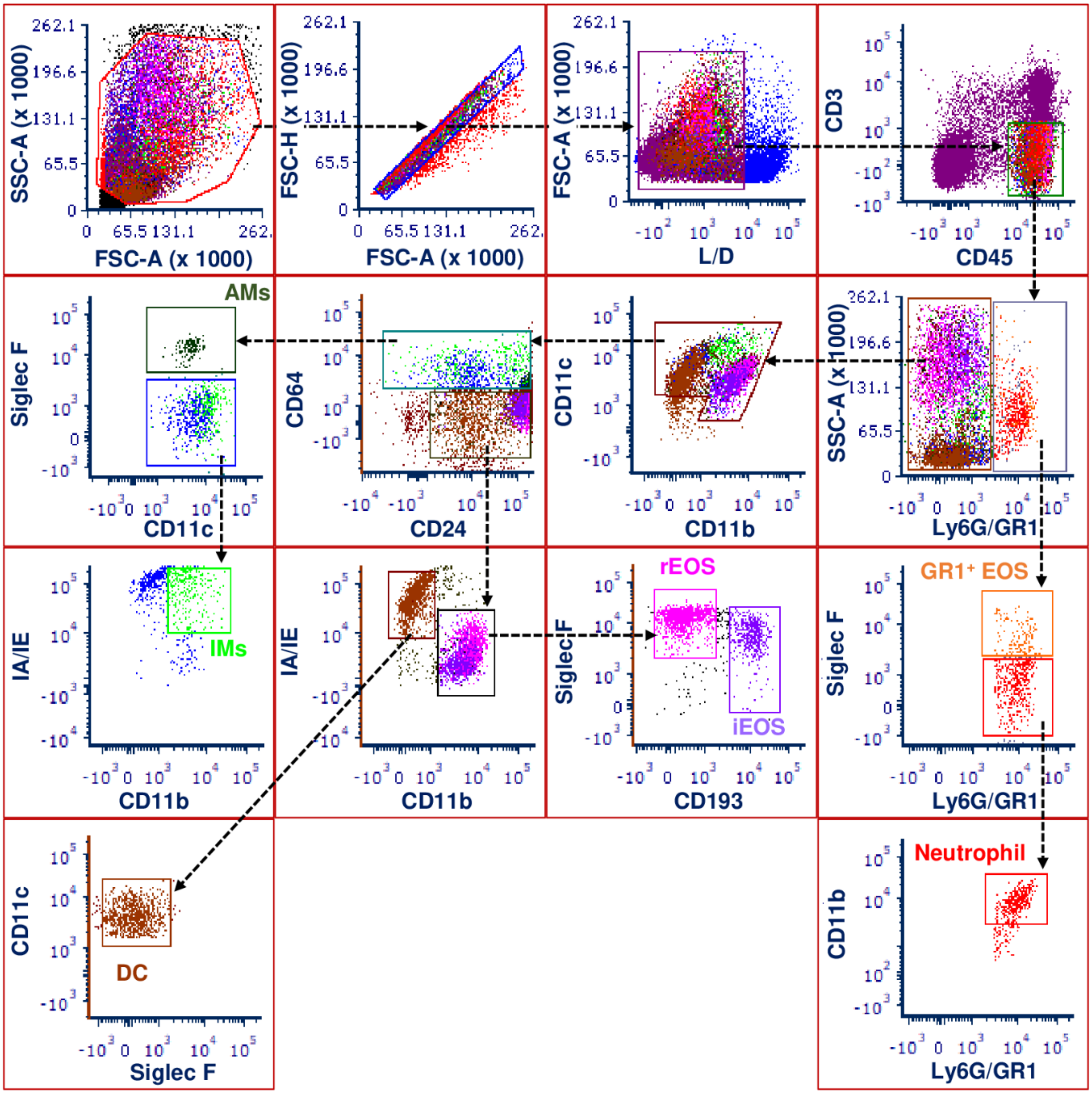

Figure S2. Representative dot plots of a multicolor flow cytometry panel used to identify myeloid cell subsets in chronic HDM model; related to Figure 1. Bronchoalveolar lavage fluid of chronic PBS and HDM (5 days/week for a total of 5 consecutive weeks) exposed mice at ZT0 and ZT12 were analyzed by flow cytometry. A custom design validated multicolor (11-color) antibody panel was used after singlet and dead cell exclusion to identify myeloid cell subsets such as mononuclear phagocytic cells (macrophages, dendritic cells) and granulocytes (neutrophils and eosinophil subtypes) in this study.

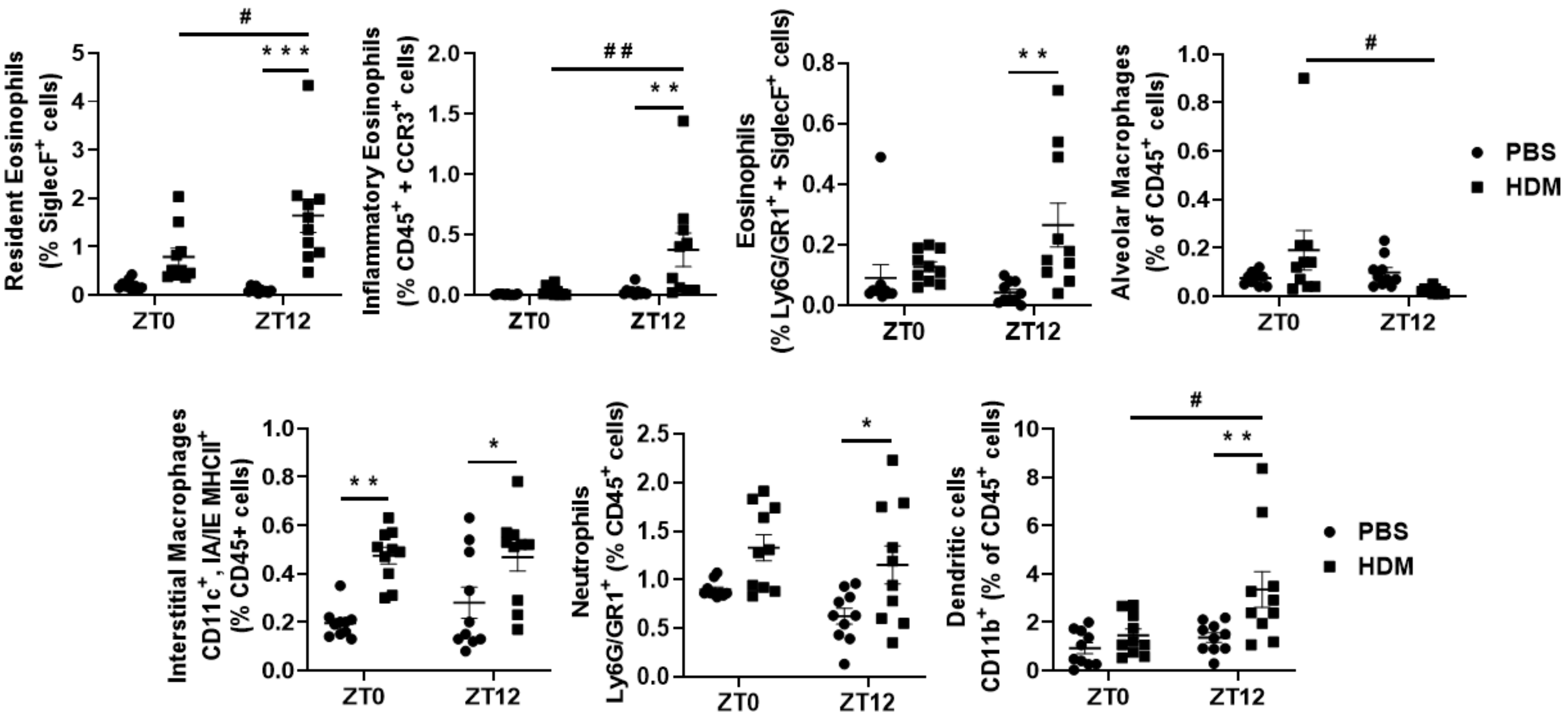

Figure S3. Myeloid cell infiltration in the lung shows a time-of-day response to chronic HDM exposure; related to Figure 1. Myeloid cell types: rEOS, iEos, Ly6G/GR1$^+$Eos, AMs, IMs, neutrophils, and DCs from lung tissue of chronic (5 days/week for 5 weeks) PBS and HDM-exposed mice (combined females and males) at ZT0 and ZT12 were analyzed by flow cytometry. Data are shown as mean ± SEM, Two-way ANOVA followed by Tukey's multiple comparison test (n=10/group [combined females and males]). **P* < 0.05, ***P* < 0.01, ****P* < 0.001, compared to respective control (PBS) at ZT0 or ZT12; $^{\#}$ *P* < 0.05, $^{\#\#}$ *P* < 0.01, compared to HDM at ZT0 vs. ZT12.

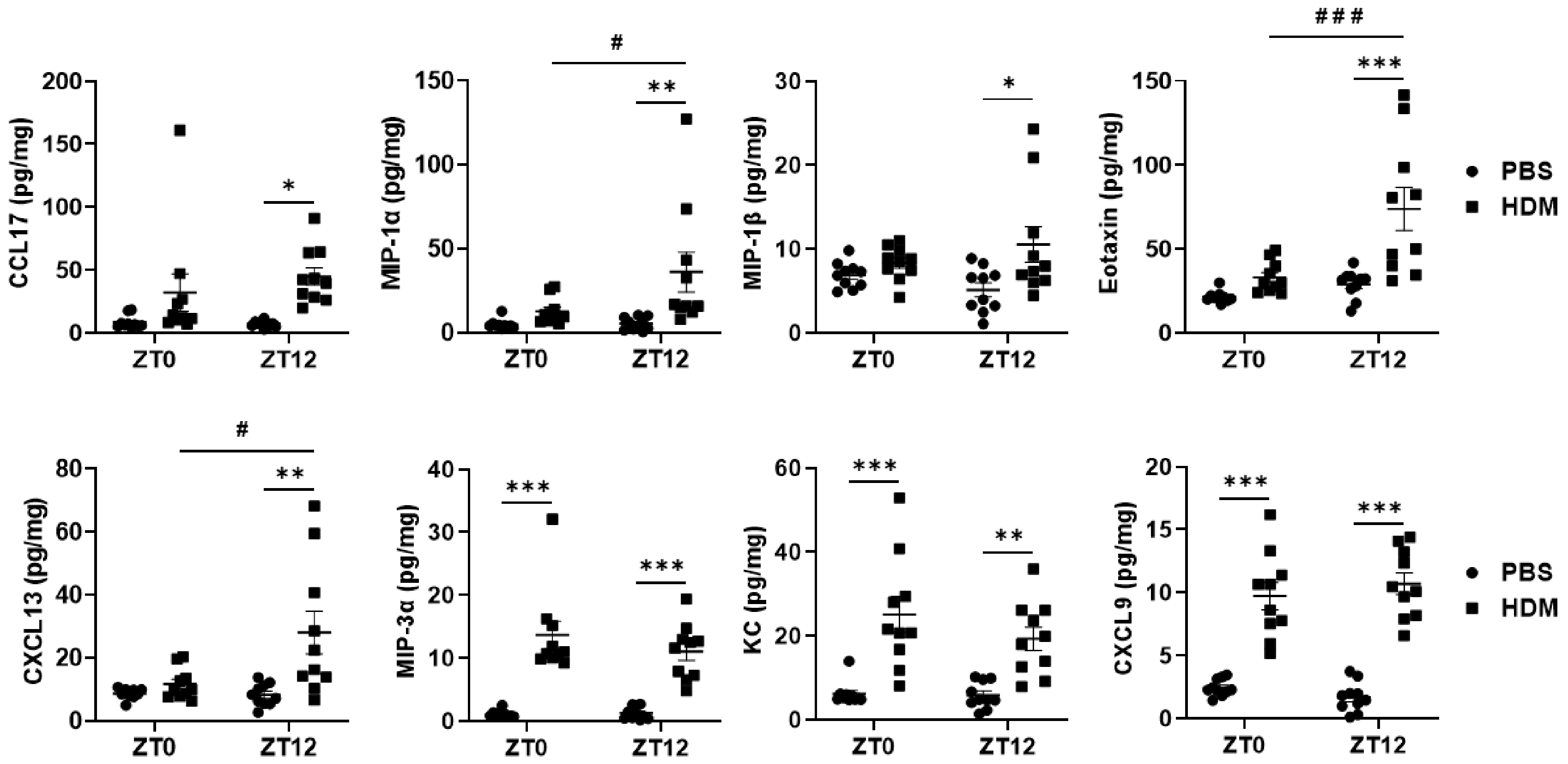

Figure S4. Lung chemokines show a time-of-day response to chronic HDM exposure; related to Figure 2. Chemokines in lung homogenates of chronic (5 days/week for 5 weeks) PBS and HDM-exposed mice (combined females and males) at ZT0 and ZT12 were analyzed using LEGENDplex Mouse Proinflammatory Chemokine Panel (13-plex), based on cytometric bead array method. Data are shown as mean ± SEM, Two-way ANOVA followed by Tukey's multiple comparison test (n=10/group [combined females and males]). $^{*}P < 0.05$, $^{**}P < 0.01$, $^{***}P < 0.001$, compared to respective control (PBS) at ZT0 or ZT12; $^{\#}P < 0.05$, $^{\#\#\#}P < 0.001$, compared to HDM at ZT0 vs. ZT12.

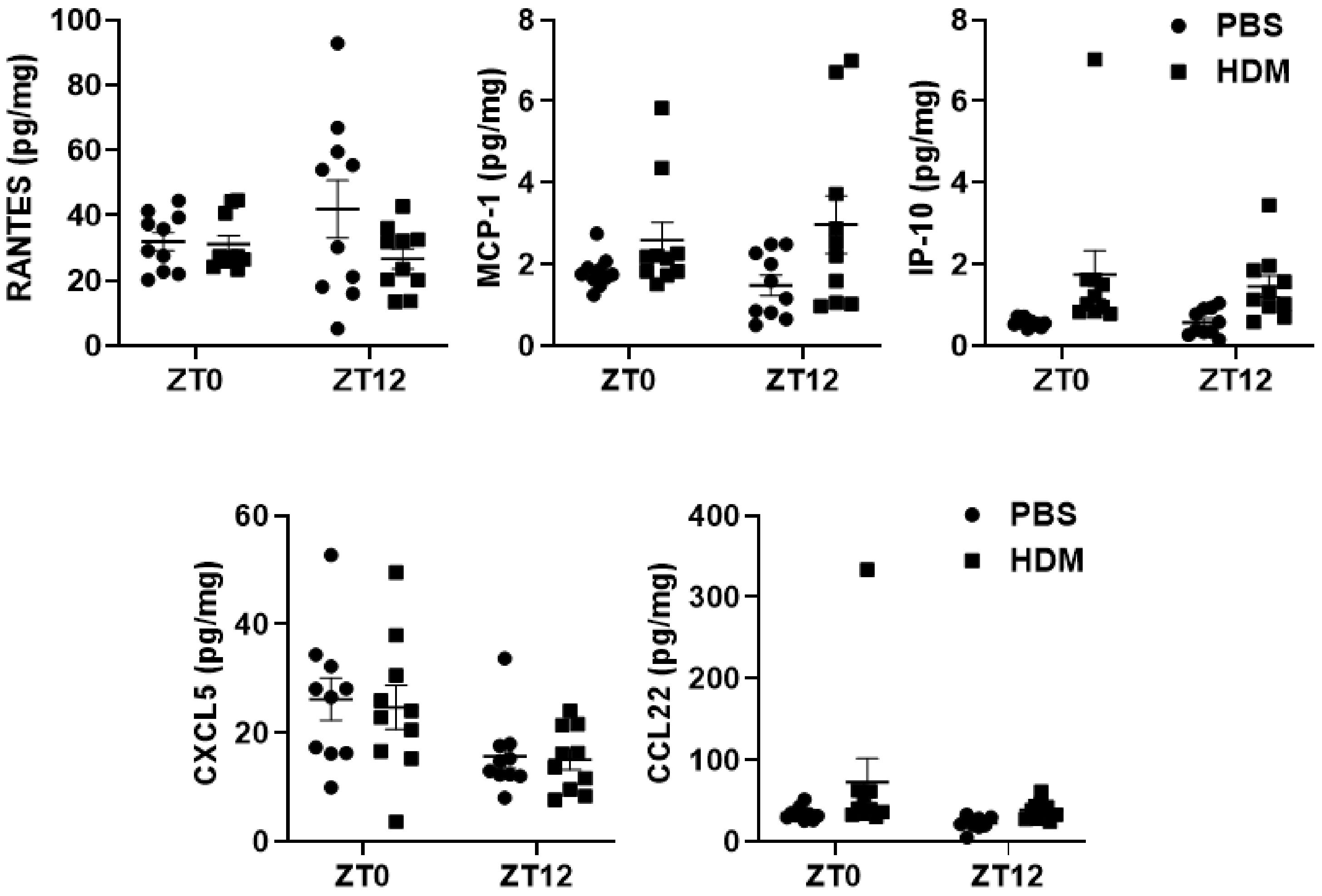

Figure S5. Lung chemokines that show no time-of-day response to chronic HDM exposure; related to Figure 2. Chemokines in lung homogenates of chronic (5 days/week for 5 weeks) PBS and HDM-exposed mice (combined females and males) at ZT0 and ZT12 were analyzed using LEGENDplex Mouse Proinflammatory Chemokine Panel (13-plex), based on cytometric bead array method. Data are shown as mean ± SEM, Two-way ANOVA followed by Tukey's multiple comparison test (n=10/group [combined females and males]).

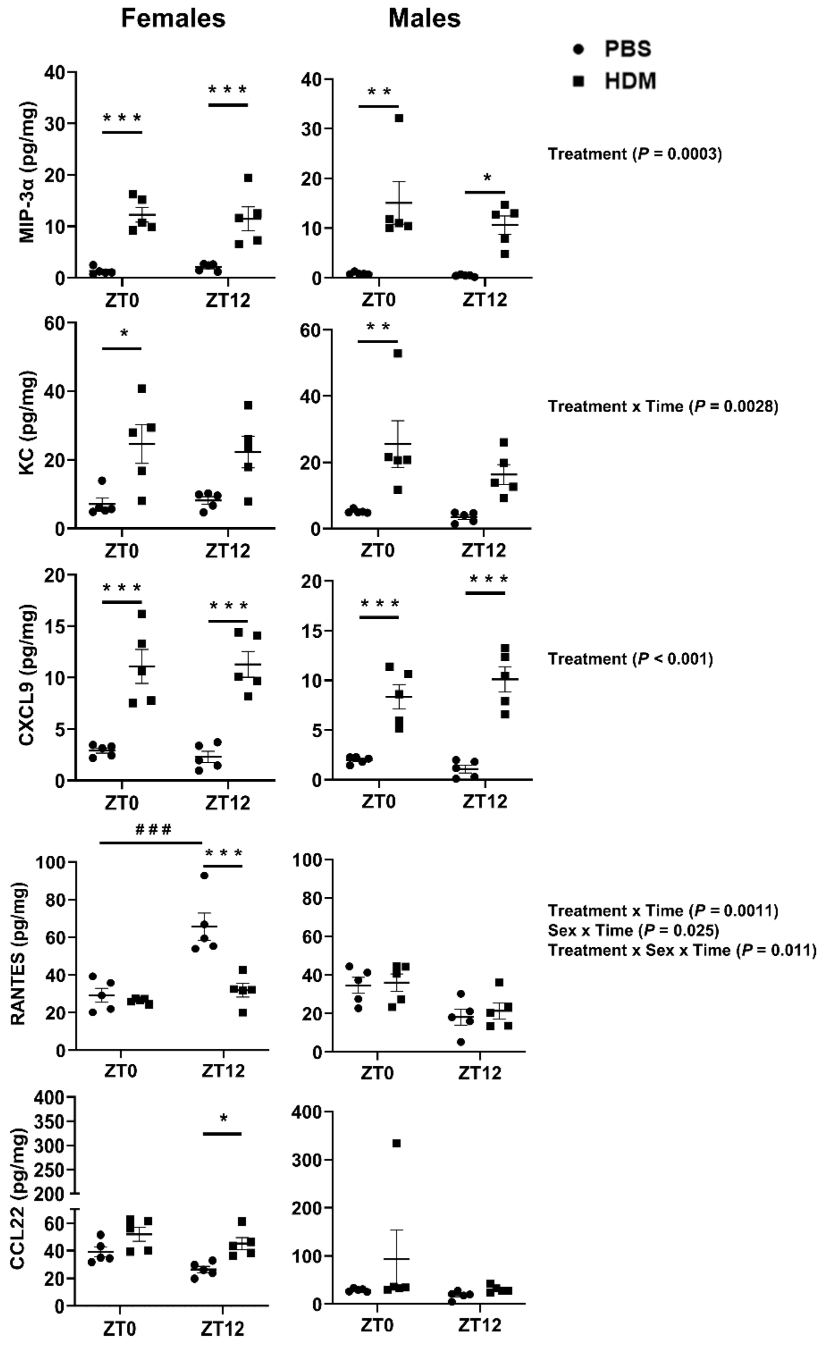

Figure S6. Lung proinflammatory chemokines show no time-of-day response and sex-based difference to chronic HDM exposure; related to Figure 2 and Table S3. Chemokines in lung homogenates of chronic (5 days/week for 5 weeks) PBS and HDM-exposed females and males at ZT0 and ZT12 were analyzed using LEGENDplex Mouse Proinflammatory Chemokine Panel (13-plex), based on cytometric bead array method. Data are shown as mean ± SEM, Two-way ANOVA followed by Tukey's multiple comparison test (n=5/group [females and males]). $^{*}P < 0.05$, $^{**}P < 0.01$, $^{***}P < 0.001$, compared to respective control (PBS) at ZT0 or ZT12; $^{\#\#\#}P < 0.001$, compared to HDM at ZT0 vs. ZT12.Summary statistics for interaction between Treatment x Sex x Time were analyzed using generalized linear modeling using R (see **Table S3**).

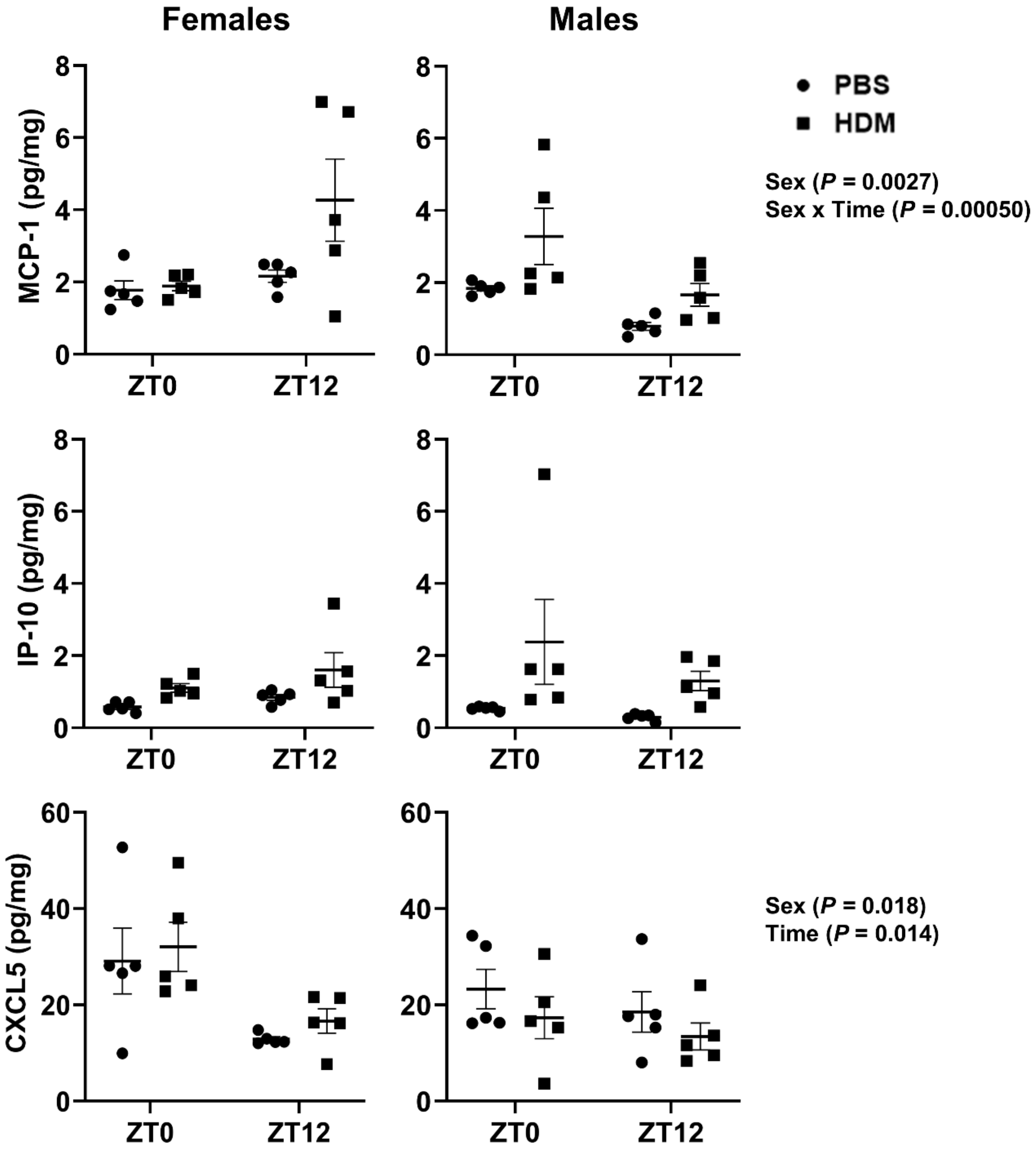

Figure S7. Lung chemokines that show no time-of-day response to chronic HDM exposure in female and male mice; related to Figure 2 and Table S3. Chemokines in lung homogenates of chronic (5 days/week for 5 weeks) PBS and HDM-exposed females and males at ZT0 and ZT12 were analyzed using LEGENDplex Mouse Proinflammatory Chemokine Panel (13-plex), based on cytometric bead array method. Data are shown as mean ± SEM, Two-way ANOVA followed by Tukey's multiple comparison test (n=5/group [females and males]). Summary statistics for interaction between Treatment x Sex x Time were analyzed using generalized linear modeling using R (see **Table S3**).

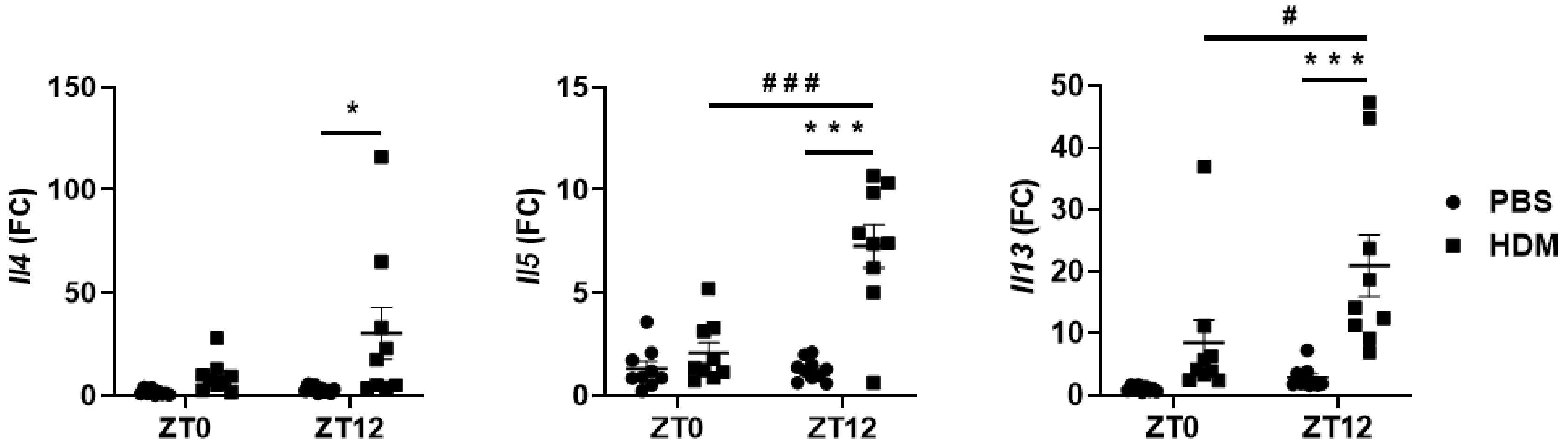

Figure S8. Gene expression of Th2 cytokines shows a time-of-day response to chronic HDM exposure; related to Figure 3. Total RNA was isolated from the lungs of chronic (5 days/week for 5 weeks) PBS and HDM-exposed mice (combined females and males) at ZT0 and ZT12. Gene expression of Th2 chemokines (*il4, il5,* and *il13*) were determined by qRT-PCR analysis relative to 18S rRNA as housekeeping control. Relative expression (fold change) was determined by the $2^{-\Delta\Delta Ct}$ method. Data are shown as mean ± SEM, Two-way ANOVA followed by Tukey's multiple comparison test (n=9-10/group [combined females and males]. **P* < 0.05, ****P* < 0.001 compared to respective control (PBS) at ZT0 or ZT12; # *P* < 0.05, ###*P* < 0.001, compared to HDM at ZT0 vs. ZT12.

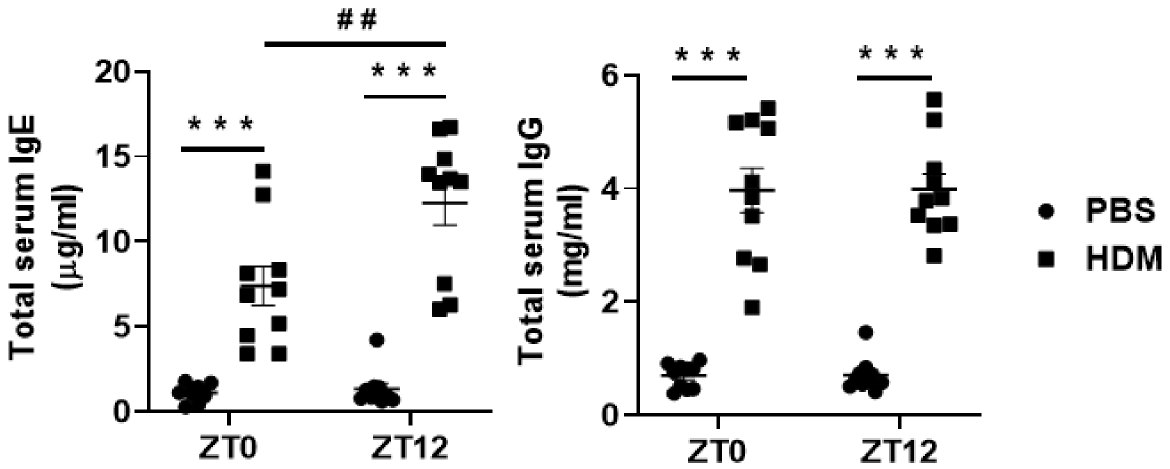

Figure S9. Serum levels of total IgE and IgG show a time-of-day response to chronic HDM exposure; related to Figure 4. Total IgE and IgG in the serum of chronic (5 days/week for 5 weeks) PBS and HDM-exposed mice (combined females and males) at ZT0 and ZT12 were determined by ELISA. Data were expressed as ng/ml and mg/ml for IgE and IgG, respectively. Data are shown as mean ± SEM, Two-way ANOVA followed by Tukey's multiple comparison test (n=10/group [combined females and males]). ****P* < 0.001, compared to respective control (PBS) at ZT0 or ZT12; $^{\#\#}$ *P* < 0.01, compared to HDM at ZT0 vs. ZT12.

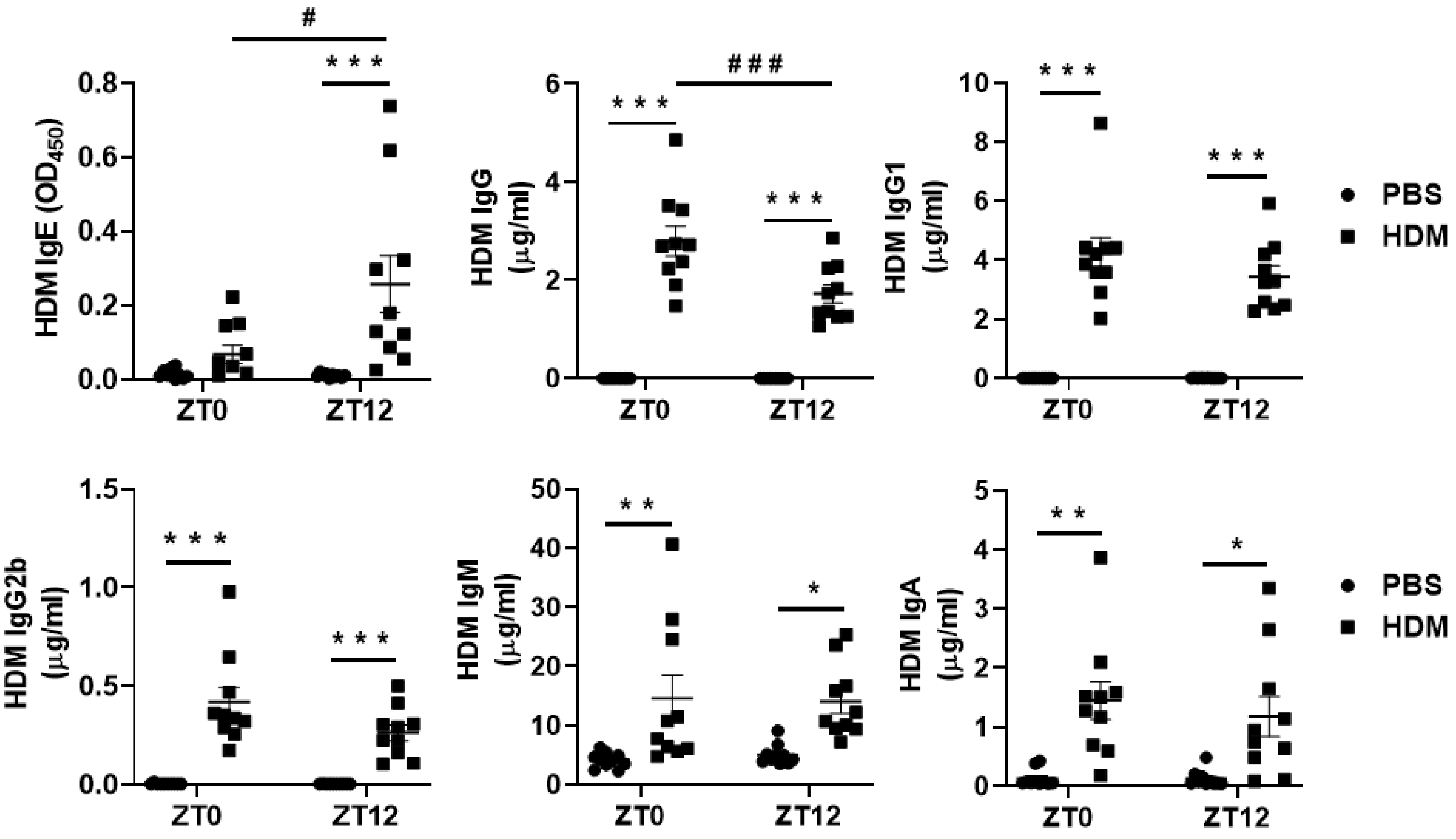

Figure S10. HDM-specific immunoglobulin responses in chronic HDM exposure; related to Figure 4. HDM-specific IgE, IgG, IgG1, IgG2b, IgM, and IgA in the serum of chronic (5 days/week for 5 weeks) PBS and HDM-exposed mice (combined females and males) at ZT0 and ZT12 were determined by commercially available ELISA kits (Chondrex, Inc.). Data were expressed as absorbance at 450 nm for HDM-specific IgE and all the other immunoglobulins data were expressed as μg/ml. Data shown as mean ± SEM, Two-way ANOVA followed by Tukey's multiple comparison test (n=10/group) *$P < 0.05$, **$P < 0.01$, ***$P < 0.001$, compared to respective control (PBS) at ZT0 or ZT12; $^{\#}P < 0.05$, $^{\#\#\#}P < 0.001$, compared to HDM at ZT0 vs. ZT12.

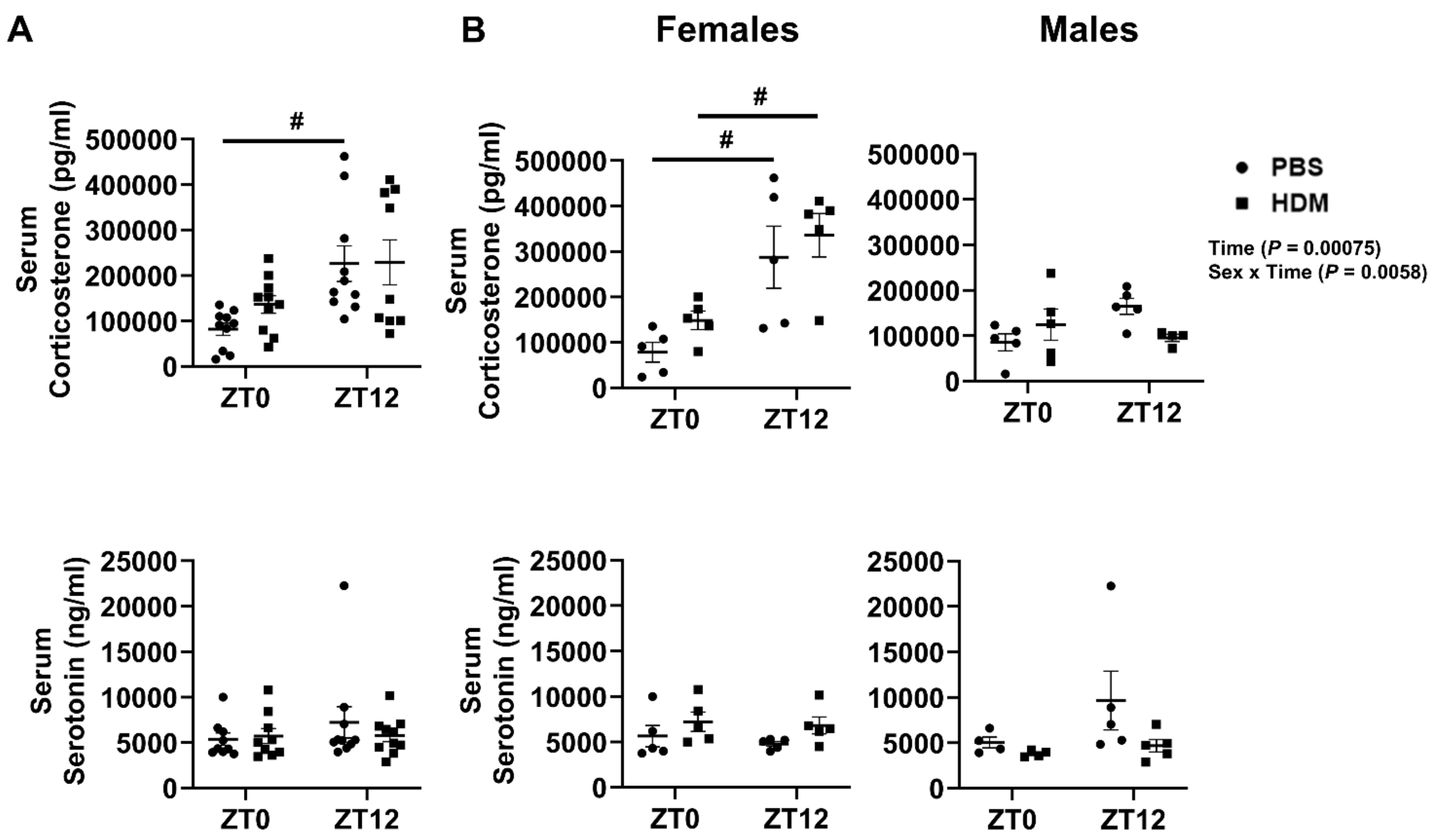

Figure S11. Stress hormones corticosterone and serotonin show differential responses to chronic PBS and HDM exposure; related to Table S3. (A) Serum levels of corticosterone and serotonin were measured in chronic PBS and HDM-exposed mice [combined females and males] at ZT0 and ZT12. **(B)** Serum levels of corticosterone and serotonin were measured in chronic PBS and HDM-exposed females at ZT0 and ZT12. **(C)** Serum levels of corticosterone and serotonin were measured in chronic PBS and HDM-exposed males at ZT0 and ZT12. Corticosterone and serotonin levels in serum were measured using commercially available ELISA kits (ENZO Life Sciences, Inc.). Data were expressed as pg/ml and ng/ml for corticosterone and serotonin, respectively. Data are shown as mean ± SEM, Two-way ANOVA followed by Tukey's multiple comparison test (n=10/group [combined females and males]; n=5/group [females and males]). # $P < 0.05$, compared to PBS or HDM at ZT0 vs. ZT12. Summary statistics for interaction between Treatment x Sex x Time were analyzed using generalized linear modeling using R (see **Table S3**).

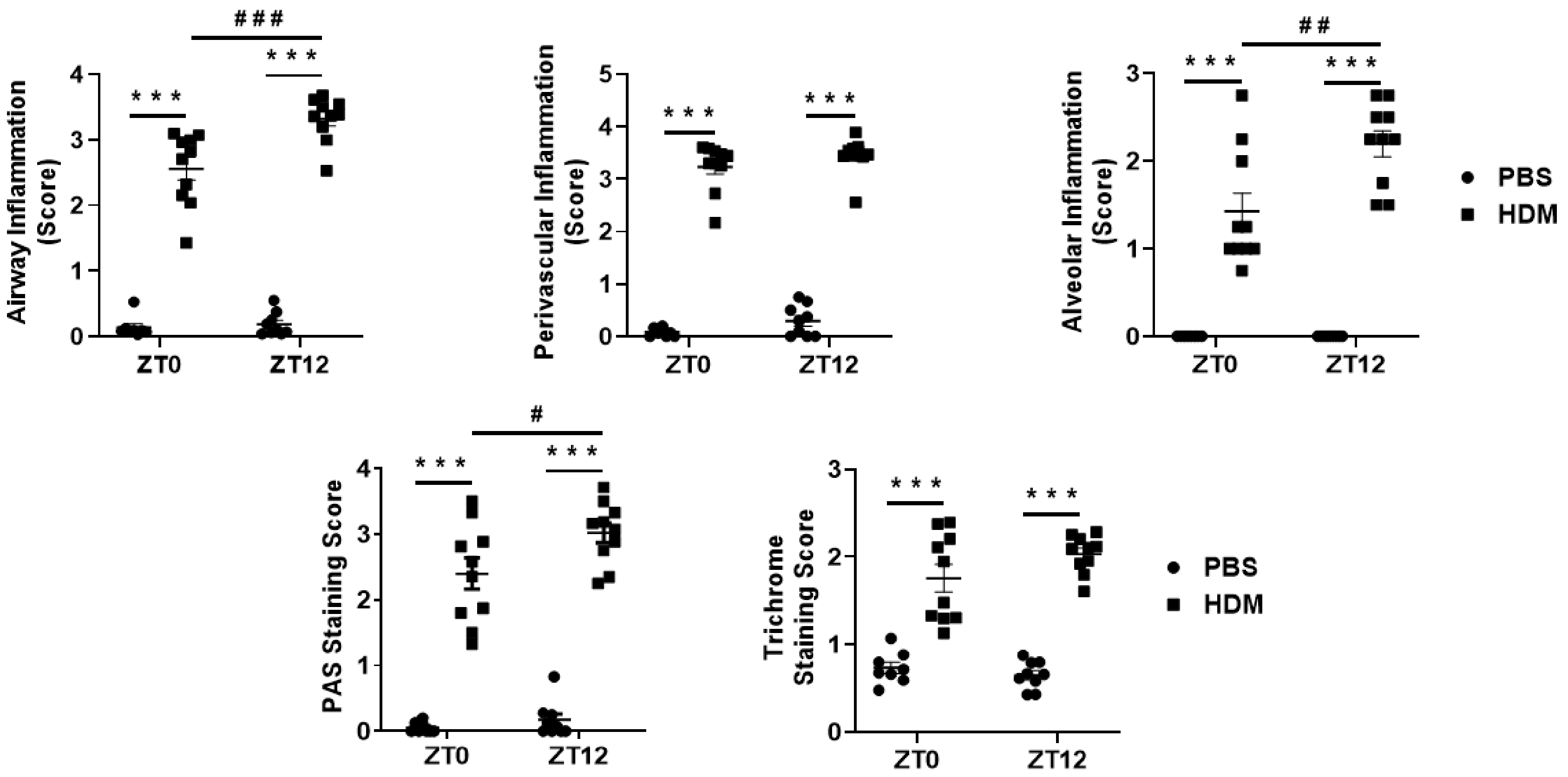

Figure S12. The histological evaluation shows a time-of-day response to the degree of lung inflammation, mucus production, and collagen deposition in chronic HDM exposure; related to Figure 5. Lung Inflammation (airway, perivascular and alveolar inflammation scores), mucus production (PAS staining score), and matrix accumulation/collagen deposition (Trichrome staining score) in mice (combined females and males) from chronic (5 days/week for 5 weeks) PBS and HDM-exposed mice at ZT0 and ZT12 were analyzed. Data are shown as mean ± SEM, Two-way ANOVA followed by Tukey's multiple comparison test (n=8 [PBS ZT0], n=10 [HDM ZT0], n=9 [PBS ZT12], and n=10 [HDM ZT12]; (combined females and males). ***$P < 0.001$, compared to respective control (PBS) at ZT0 or ZT12; $^{\#}P < 0.05$, $^{\#\#}P < 0.01$, $^{\#\#\#}P < 0.001$, compared to HDM at ZT0 vs. ZT12.

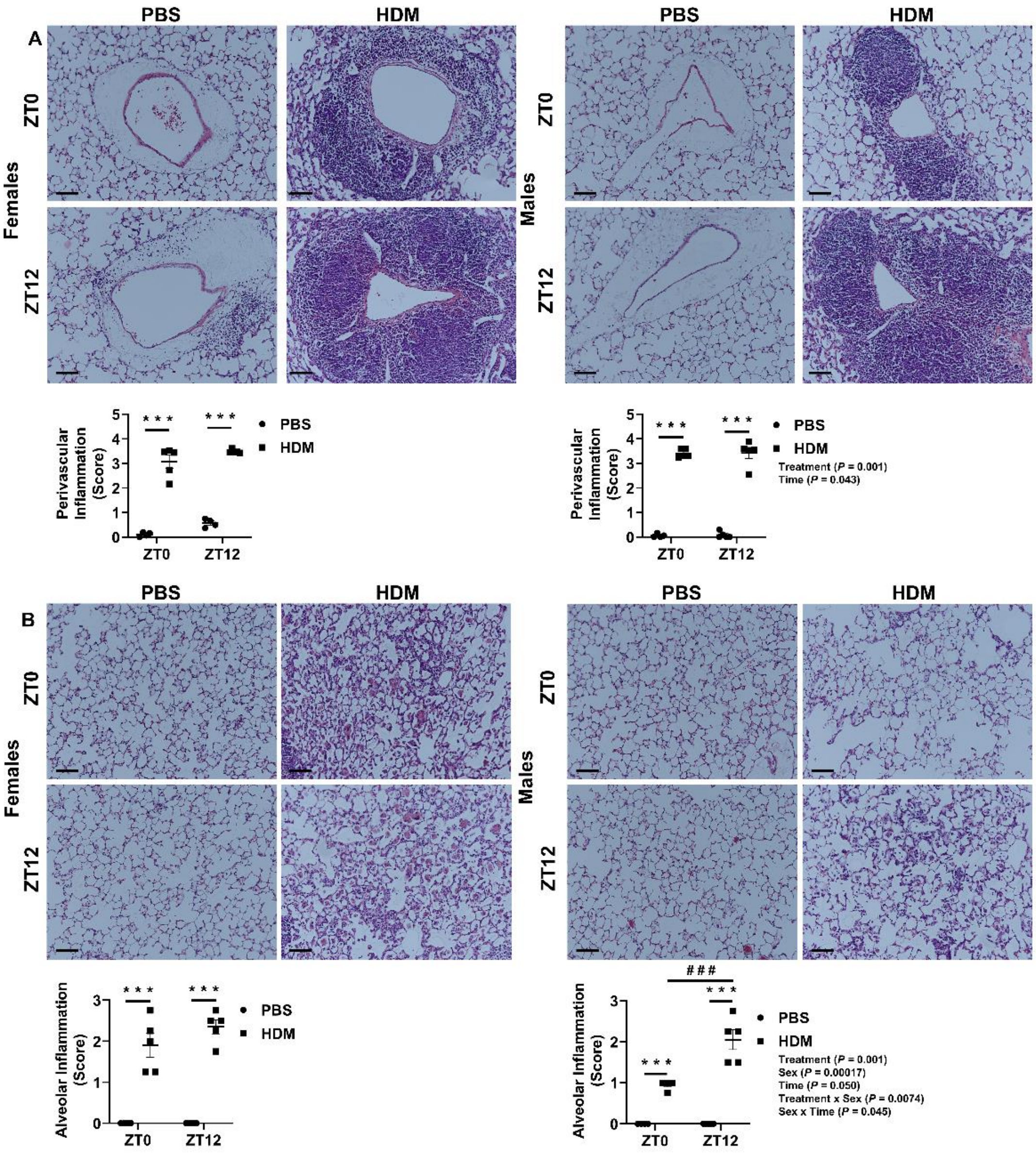

Figure S13. The histological evaluation shows a difference in the degree of perivascular and lung alveolar inflammation in chronic HDM-exposed mice; related to Figure 5 and Table S3. (A) Representative Hematoxylin and Eosin (H&E) stained lung tissue sections showed a difference in the degree of lung perivascular inflammation from chronic (5 days/week for 5 weeks) PBS and HDM-exposed females and males at ZT0 and ZT12 **(B)** Representative Hematoxylin and Eosin (H&E) stained lung tissue sections showed a difference in the degree of lung alveolar inflammation from chronic (5 days/week for 5 weeks) PBS and HDM-exposed females and males at ZT0 and ZT12 were provided. The graph shows the average perivascular and lung alveolar inflammation scores from different regions were determined using the scoring criteria in a blinded manner as described (see Materials and methods section). Scale bar (100 µm). Data are shown as mean ± SEM, Two-way ANOVA followed by Tukey's multiple comparison test (n=4-5/group [females and males]). ****P* < 0.001, compared to respective control (PBS) at ZT0 or ZT12. Summary statistics for interaction between Treatment x Sex x Time were analyzed using generalized linear modeling using R (see **Table S3**).

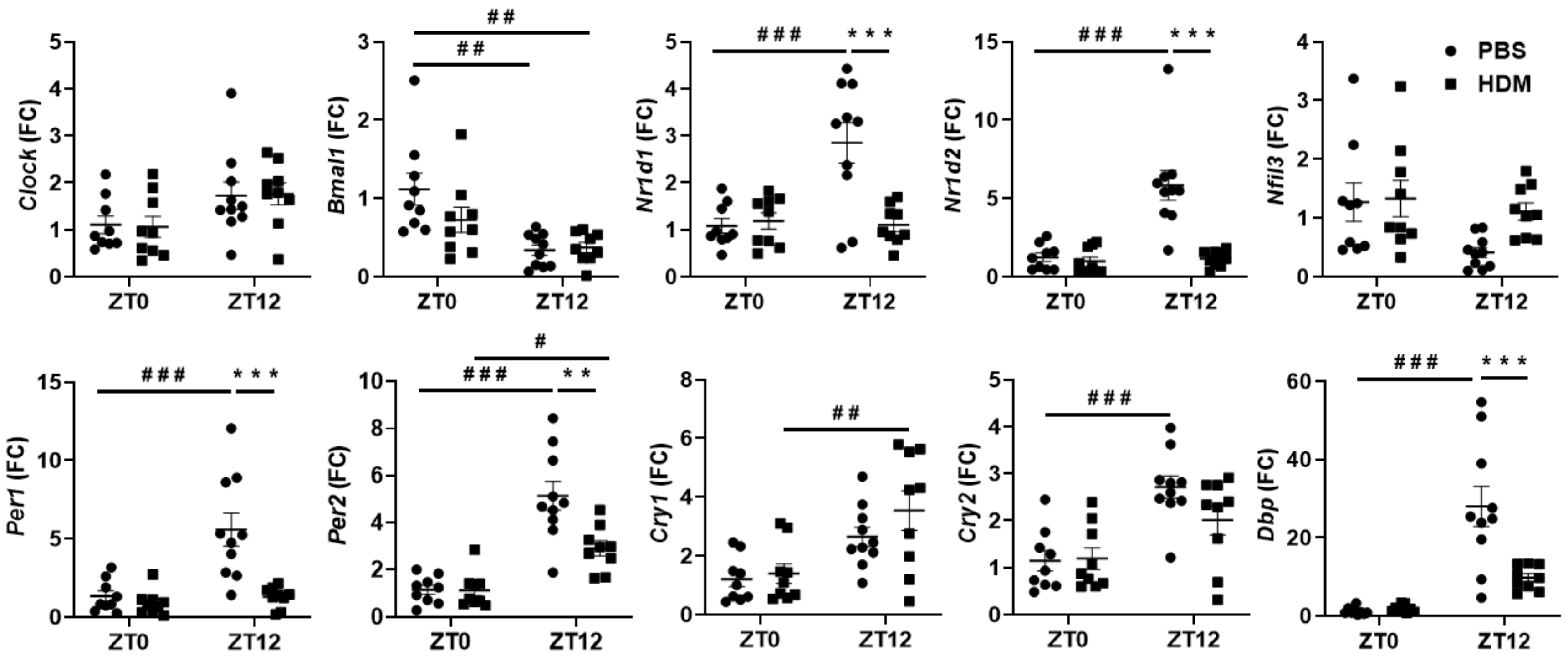

Figure S14. Gene expression of circadian clock genes shows a time-of-day response to chronic HDM exposure; related to Figure 6. Total RNA was isolated from the lungs of chronic (5 days/week for 5 weeks) PBS and HDM-exposed mice (combined females and males) at ZT0 and ZT12. Gene expression of core clock-controlled genes (*clock, bmal1, nr1d1, nr1d2, nfil3, per1, per2, cry1, cry2,* and *dbp*) were determined by qRT-PCR analysis relative to 18S rRNA as housekeeping control. Relative expression (FC: fold-change) was determined by the $2^{-\Delta\Delta Ct}$ method. Data are shown as mean ± SEM, Two-way ANOVA followed by Tukey's multiple comparison test (n=9-10/group [combined females and males]). $^{**}P < 0.01$, $^{***}P < 0.001$ compared to respective control (PBS) at ZT0 or ZT12; $^{\#}P < 0.05$, $^{\#\#}P < 0.01$, $^{\#\#\#}P < 0.001$, compared to PBS or HDM at ZT0 vs. ZT12.

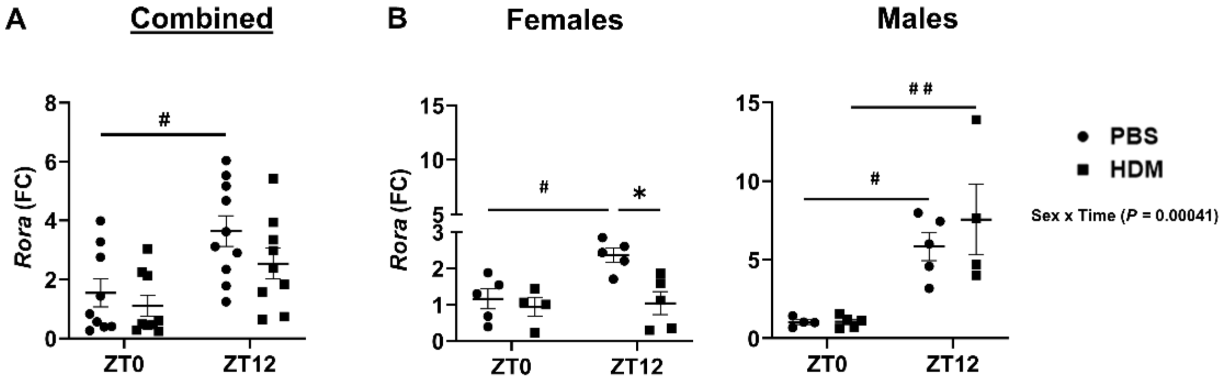

Figure S15. Gene expression of circadian gene *Rora* shows a time-of-day response to chronic HDM exposure; related to Figure 6 and Table S3. **(A)** Total RNA was isolated from the lungs of chronic (5 days/week for 5 weeks) PBS and HDM-exposed combined female and male mice at ZT0 and ZT12. **(B)** Total RNA was isolated from the lungs of chronic (5 days/week for 5 weeks) PBS and HDM-exposed females and males at ZT0 and ZT12. Gene expression of *Rora* was determined by qRT-PCR analysis relative to 18S rRNA as housekeeping control. Relative expression (FC: fold-change) was determined by the $2^{-\Delta\Delta Ct}$ method. Data are shown as mean ± SEM, Two-way ANOVA followed by Tukey's multiple comparison test (n=9-10/group [combined females and males]; n=4-5/group [females and males]).). $^{**}P < 0.01$, $^{***}P < 0.001$ compared to respective control (PBS) at ZT0 or ZT12; $^{\#}P < 0.05$, $^{\#\#}P < 0.01$, $^{\#\#\#}P < 0.001$, compared to PBS or HDM at ZT0 vs. ZT12. Summary statistics for interaction between Treatment x Sex x Time were analyzed using generalized linear modeling using R (see **Table S3**).

Table S1. Mouse gene-specific qRT-PCR primers used in this study; related to the STAR Methods

Gene symbol	Forward Primer Sequence (5'-3')	Reverse Primer Sequence (5'-3')
Il4	5'-GGTGTTCTTCGTTGCTGTGA-3'	5'-TCTCGAATGTACCAGGAGCC-3'
Il5	5'-AGCCCCTGAAAGATTTCTCC-3'	5'-ATGGAGATTCCCATGAGCAC-3'
Il13	5'-ATGTTGGTCAGGGAATCCAG-3'	5'-TGTGTCTCTCCCTCTGACCC-3'
Clock	5'-GGAGTCTCCAACACCCACAG-3'	5'-GGCACGTGAAAGAAAAGCAC-3'
Bmal1	5'-AAGGGCCACTGTAGTTGCTG-3'	5'-CTGCAGTGAATGCTTTTGGA-3'
Nr1d1	5'-GAGTCAGGGACTGGAAGCTG-3'	5'-AAGACATGACGACCCTGGAC-3'
Nr1d2	5'-TGGAGGCAGAGCTAGAGGAA-3'	5'-ACCCGGTGCTCATGATGT-3'
Nfil3	5'-GAACTCTGCCTTAGCTGAGGT-3'	5'-ATTCCCGTTTTCTCCGACACG-3'
Per1	5'-AACGCTTTGCTTTAGATCGG-3'	5'-TCCTCAACCGCTTCAGAGAT-3'
Per2	5'-CTTGGGGAGAAGTCCACGTA-3'	5'-TACTGGGACTAGCGGCTCC-3'
Cry1	5'-GTCCCCGAATCACAAACAGA-3'	5'-TGCGTCTATATCCTCGACCC-3'
Cry2	5'-TCCCCGGACTACAAACAGAC-3'	5'-GTCTACATCCTCGACCCGTG-3'
Dbp	5'-ATTAGCACCTCCACGGTGTC-3'	5'-GGCTCTTGCAGCTCCTCTT-3'
Rora	5'-TTGCAGCCTTCACACGTAAT-3'	5'-AGGCAGAGCTATGCGAGC-3'
Rn18s	5'-GTAACCCGTTGAACCCCATT-3'	5'-CCATCCAATCGGTAGTAGCG-3'

Table S4. Chronic HDM-induced T helper cytokines analyzed in lung homogenates from females and males

	Female				Male			
	ZT0		ZT12		ZT0		ZT12	
**Analytes (pg/mg)**	**PBS**	**HDM**	**PBS**	**HDM**	**PBS**	**HDM**	**PBS**	**HDM**
**IFN-γ**	0.86 ± 0.13	1.92 ± 0.74	0.57 ± 0.17	2.70 ± 2.18	0.41 ± 0.04	2.09 ± 0.66	0.37 ± 0.05	1.84 ± 0.51
**IL-5**	0.53 ± 0.19	0.40 ± 0.04	0.40 ± 0.12	1.87 ± 0.55	0.17 ± 0.02	2.98 ± 2.57	0.22 ± 0.01	0.28 ± 0.05
**TNF-α**	2.01 ± 0.36	2.34 ± 0.57	3.52 ± 0.40	5.60 ± 1.66	1.32 ± 0.15	3.20 ± 0.66	1.15 ± 0.22	4.00 ± 0.91
**IL-2**	26.80 ± 3.86	9.89 ± 2.81[a]	21.87 ± 2.33	6.03 ± 2.56 [b]	20.18 ± 2.85	14.80 ± 2.45	13.89 ± 4.68	7.54 ± 2.07
**IL-6**	4.72 ± 1.45	3.78 ± 0.96	4.66 ± 1.63	3.25 ± 0.78	4.02 ± 1.19	3.92 ± 0.82	5.27 ± 1.56	2.52 ± 0.50
**IL-4**	0.46 ± 0.13	1.24 ± 0.49	0.38 ± 0.06	6.98 ± 4.21	0.18 ± 0.02	0.86 ± 0.39	0.17 ± 0.01	1.05 ± 0.51
**IL-10**	3.02 ± 1.15	0.88 ± 0.86	1.51 ± 0.92	2.01 ± 0.75	0.03 ± 0.00	0.53 ± 0.37	0.03 ± 0.00	0.04 ± 0.02
**IL-9**	12.98 ± 1.83	4.62 ± 0.44[c]	5.75 ± 0.42 [c]	5.19 ± 1.13	5.63 ± 0.17	6.52 ± 1.23	5.24 ± 0.51	3.76 ± 0.81
**IL-17A**	6.45 ± 1.17	13.21 ± 7.33	2.84 ± 1.06	20.14 ± 7.10	1.40 ± 0.28	10.19 ± 5.94	1.08 ± 0.18	19.01 ± 4.89
**IL-17F**	0.58 ± 0.14	0.26 ± 0.09	0.29 ± 0.08	0.34 ± 0.05	0.20 ± 0.02	0.35 ± 0.14	0.20 ± 0.03	0.21 ± 0.03
**IL-22**	0.79 ± 0.18	0.17 ± 0.03	0.51 ± 0.17	0.54 ± 0.18	0.30 ± 0.05	3.03 ± 2.57	0.29 ± 0.02	0.22 ± 0.04
**IL-13**	1.97 ± 0.34 [b]	0.73 ± 0.17	1.01 ± 0.29	1.55 ± 0.28	0.82 ± 0.12	3.07 ± 2.13	0.58 ± 0.06	0.72 ± 0.28

Data shown as mean ± SEM, Two-way ANOVA followed by Tukey's multiple comparison test (n=5/group); [a] $P < 0.01$ compared to ZT0 PBS; [b]$P < 0.02$ compared to ZT12 PBS; [c]$P < 0.001$ compared to ZT0 PBS.

Table S5. Chronic HDM-induced proinflammatory cytokines analyzed in lung homogenates from females and males (combined)

	Female + Male (Combined)			
	ZT0		ZT12	
**Analytes (pg/mg)**	**PBS**	**HDM**	**PBS**	**HDM**
**IFN-γ**	0.64 ± 0.10	2.01 ± 0.47	0.47 ± 0.09	2.27 ± 1.06
**IL-5**	0.35 ± 0.11	1.69 ± 1.28	0.31 ± 0.06	1.07 ± 0.37
**TNF-α**	1.66 ± 0.22	2.77 ± 0.44	2.33 ± 0.45	4.80 ± 0.93 [a]
**IL-2**	23.49 ± 2.52	12.34 ± 1.94 [b]	17.88 ± 2.80	6.78 ± 1.57 [c]
**IL-6**	4.37 ± 0.89	3.85 ± 0.59	4.96 ± 1.07	2.88 ± 0.45
**IL-4**	0.32 ± 0.08	1.05 ± 0.30	0.28 ± 0.04	4.01 ± 2.23
**IL-10**	1.52 ± 0.74	0.70 ± 0.45	0.77 ± 0.50	1.02 ± 0.48
**IL-9**	9.30 ± 1.50	5.57 ± 0.69 [d]	5.49 ± 0.32 [d]	4.47 ± 0.70
**IL-17A**	3.93 ± 1.01	11.70 ± 4.48	1.96 ± 0.59	19.57 ± 4.07[e]
**IL-17F**	0.39 ± 0.09	0.31 ± 0.08	0.25 ± 0.04	0.27 ±0.04
**IL-22**	0.55 ± 0.12	1.60 ± 1.30	0.40 ± 0.09	0.38 ± 0.10
**IL-13**	1.39 ± 0.26	1.90 ± 1.08	0.80 ± 0.16	1.13 ± 0.23

Data shown as mean ± SEM, Two-way ANOVA followed by Tukey's multiple comparison test (n=10/group); [a]$P < 0.02$ compared to ZT12 PBS; [b]$P < 0.007$ compared to ZT0 PBS; [c]$P < 0.007$ compared to ZT12 PBS; [d]$P < 0.03$ compared to ZT0 PBS; [e]$P < 0.001$ compared to ZT12 PBS.
